# Oral Microbial Determinants of Saliva and Serum Lipopolysaccharide Activity

**DOI:** 10.1177/00220345251370995

**Published:** 2025-10-18

**Authors:** M. Manzoor, J. Putaala, S. Zaric, J. Leskelä, A. Dong, E. Könönen, L. Lahti, S. Paju, P. J. Pussinen

**Affiliations:** 1 Department of Oral and Maxillofacial Diseases, University of Helsinki, Helsinki, Finland; 2 Neurology, Helsinki University Hospital and University of Helsinki, Helsinki, Finland; 3 Centre for Host-Microbiome Interactions, Faculty of Dentistry, Oral & Craniofacial Sciences, King’s College London, UK; 4 Institute of Dentistry, University of Turku, Turku, Finland; 5 Department of Computing, University of Turku, Turku, Finland; 6 School of Medicine, Institute of Dentistry, University of Eastern Finland, Kuopio, Finland

**Keywords:** oral microbiome, metagenomic sequencing, gram-negative bacteria, endotoxemia, biomarkers, periodontitis

## Abstract

Lipopolysaccharide (LPS) is a virulence factor of gram-negative bacteria, and endotoxemia or translocation of LPS in serum plays a significant role in oral and systemic pathologies. The contribution of the oral microbiome composition to saliva LPS activity and endotoxemia remains unclear. We investigated whether salivary and serum LPS levels are associated with oral microbiome diversity, taxonomic profiles, and functional characteristics. The oral microbiome was analyzed using metagenomic sequencing of saliva from 298 individuals enrolled in a multicenter case-control study, SECRETO (NCT01934725). Serum and salivary LPS activities were measured, and multiple linear regression models were fitted to identify the microbial taxa that predicted LPS levels. MaAsLin2 (Microbiome Multivariable Associations with Linear Models) was used to determine the associations of microbial functional features and LPS levels. Salivary alpha diversity was positively associated with serum LPS but negatively associated with salivary LPS, smoking, and antibiotic use in the preceding 1 to 6 mo. Community composition (beta diversity) differed between the salivary LPS tertiles (*P* = 0.001) but not between serum LPS tertiles. In total, 10 oral taxa associated with serum LPS tertiles and 59 with salivary LPS tertiles were identified. *Prevotella*, *Neisseria*, *Leptotrichia*, and *Porphyromonas* had significant positive associations with salivary LPS, whereas *Fusobacterium* had a negative association. Among these genera, *Prevotella* sp. E13_17, *P. gingivalis*, *L. wadei*, and *F. nucleatum* were the species with the strongest associations. Among the 1,016 oral microbiome metabolic features, several were linked to the biosynthesis of LPS, lipid A, and O-antigen pathways. The oral microbiome composition was strongly associated with salivary LPS activity in addition to weaker links to serum LPS. Oral microbiota–derived LPS activity in saliva was associated with microbial metabolism characterized by the predominance of proliferation and biosynthesis pathways. Our study indicates that dysbiosis of the oral microbiome is a source of increased salivary and serum LPS activity.

## Introduction

Lipopolysaccharide (LPS) is a crucial virulence factor of gram-negative bacteria, constituting 10% to 15% of bacterial outer membrane molecules and accounting for up to 75% of its total surface area (Erridge et al. 2002). The primary role of LPS is to preserve the cell shape and structural integrity of the outer membrane, as the cellular wall is the first line of defense against the immune system of the host. LPS consists of 3 distinct domains, namely, O-antigen, the immunodominant, hydrophilic portion pointing out of the cell membrane; the core oligosaccharide, embedded in the cytoplasmic side of the inner membrane and influencing cell wall phenotype, poring, efflux, and responses against the environment; and lipid A, which is anchored in the outer membrane and is the primary immunostimulatory component of LPS (Valvano 2015).

LPS quantification is based on the biological activity of the lipid A portion of the molecule. The gold standard method still relies on the *Limulus* amebocyte lysate assay (LAL) and its ability to agglutinate upon encountering LPS. Thus, determining LPS concentration is based on an indirect assay that measures host innate immunity response, namely, the amebocytes enzymatic cascade triggered by LPS and eventually resulting in coagulation, immobilization, and neutralization of the invading gram-negative bacteria (Munford 2016).

Translocation of LPS to the bloodstream causes endotoxemia, which is associated with low-grade inflammation, activation of the complement system and the contact activation pathway, and metabolic alterations (Pussinen et al. 2022). Accordingly, LPS may be a potent molecular mediator between periodontitis and cardiometabolic disorders (Liljestrand et al. 2017), especially since LPS may enter the circulation via inflamed periodontal tissues (Geerts et al. 2002; Lockhart et al. 2008). Severe forms of periodontitis with a high grade of progression are associated with elevated serum LPS levels (Shaddox et al. 2011; Leira et al. 2021), which decrease after periodontal treatment (Pussinen et al. 2004). Although the gut microbiome has long been considered the main source of endotoxemia, the oral microbiome may also contribute to the systemic inflammatory burden (Mukherjee et al. 2025). Furthermore, the mouth is the starting point of the gastrointestinal tract, and the 2 microbiomes are continuously connected to each other. Nevertheless, the composition of the oral microbiome in endotoxemia has not been investigated previously.

In patients with unstable periodontitis, 4.9% of serum LPS variation was explained by saliva LPS levels (Liljestrand et al. 2017). Although saliva LPS may be used as a biomarker of gram-negative microbiome dominance and thus periodontitis, LPS has not been investigated as comprehensively as other bacterial biomarkers (Dong et al. 2025). Saliva LPS activity is positively associated with periodontal diagnosis (Zaric et al. 2022; Leskelä et al. 2024); alveolar bone loss; a cluster of subgingival oral, oropharyngeal, or nasopharyngeal species (Liljestrand et al. 2017); and cumulative risk score of periodontitis (Liukkonen et al. 2020). Functional profiling of the oral microbiome revealed enriched LPS biosynthesis in periodontitis and its related systemic complications (Zhao et al. 2022; Oh et al. 2023). However, no studies have directly established the composition of the oral microbiome regarding salivary LPS levels.

We investigated the composition and functional features of the oral microbiome that are associated with salivary LPS activity and endotoxemia.

## Materials and Methods

### Study Subjects and Recruitment

The present study, SECRETO Oral (Leskelä et al. 2024), is a substudy of the international, multicenter SECRETO case-control study (Putaala et al. 2017). All participants provided written informed consent before participating in the study. This study was conducted in accordance with the STROBE guidelines and the Declaration of Helsinki. This study was approved by the Helsinki and Uusimaa Hospital District (362/13/03/00/2012) and the local Ethics Committees at each recruiting center.

At the time of recruitment, all participants underwent a thorough structured interview, and a detailed clinical history was obtained from medical records.

### Clinical Oral Examination

All oral examinations were conducted between April 2014 and February 2020 by a single periodontist (S. Paju). Periodontal diagnosis was defined according to the 2017 classification (Chapple et al. 2018), and the participants were divided into 3 groups: periodontal health, gingivitis, and periodontitis. Clinical moderate or extensive caries lesions (levels 3–6) were detected and classified using the International Caries Detection and Assessment System.

### Sample Collection and Metagenomic Sequencing

The procedures for saliva sample collection, processing, DNA extraction, and sequencing have been previously described (Manzoor, Leskelä, Pietiäinen, Martinez-Majander, Könönen, et al. 2024; Manzoor, Leskelä, Pietiäinen, Martinez-Majander, Ylikotila, et al. 2024; Manzoor et al. 2025). In brief, DNA was extracted from stimulated saliva samples followed by metagenomic sequencing using an Illumina NovaSeq 6000.

### LPS measurement

LPS activity in serum was measured using the *Limulus* amebocyte lysate assay coupled with a chromogenic substrate (LAL) (Hycult Biotech, The Netherlands) and in saliva using the fluorescent recombinant Factor C assay (EndoZyme II Go Plate, bioMérieux) with dilutions of 1:5 for serum and 1:1,000 for saliva in endotoxin-free water. LPS activity within the sample type was categorized into tertiles to obtain low, medium, and high categories. The cutoff levels for serum were 0.331 EU/mL and 0.473 EU/mL and for saliva were 6,848 EU/mL and 14,453 EU/mL.

### Statistical Analysis

All analyses were performed using R (v. 4.2.2). We computed alpha diversity metrics (Shannon and Inverse Simpson indices) and explored their relationship with LPS using Spearman correlations and linear regression models. Permutational multivariate analysis of variance (PERMANOVA) tests used the Bray–Curtis dissimilarity between LPS tertiles, and the principal coordinate analysis plot was used to visualize the beta diversity at the species level. We performed linear discriminant analysis effect size (LEfSe) analysis (Segata et al. 2011) at different taxonomic levels using LPS tertiles, applying Kruskal–Wallis and Wilcoxon tests (*P* < 0.05) for significance between LPS tertiles within the 2 sample types.

Functional profiles were generated for each sample using HUMAnN 3 using the default settings (Beghini et al. 2021). Differential abundance analysis of the functional features was performed using MaAsLin 2 (Mallick et al. 2021). Multiple testing correction using the false discovery rate was applied to control for false positives.

See the supplementary methods for further details.

## Results

### Characteristics of the Study Population

A total of 298 participants were included in this study. The demographic and clinical characteristics of the participants are summarized in Table 1. Sex, age, education level, smoking history, alcohol consumption, obesity, diabetes, hypertension, bleeding on probing, periodontal pockets ≥4 mm, and periodontal health status distribution were similar across the serum and salivary LPS tertiles. History of stroke and antibiotic use during the preceding 1 to 6 mo differed between serum LPS tertiles, whereas only the prevalence of caries differed between salivary LPS tertiles.

**Table 1.** Characteristics of the Study Participants.

		Serum Tertiles (*n* = 298)				Saliva Tertiles (*n* = 298)			
Characteristic		Low (*n* = 100)	Medium (*n* = 99)	High (*n* = 99)	*P* Value	Low (*n* = 100)	Medium (*n* = 99)	High (*n* = 99)	*P* Value
Male sex		55 (55.0)	61 (61.6)	60 (60.6)	0.592	51 (51.0)	65 (65.7)	60 (60.6)	0.102
Mean (SD) age, y		40.1 (7.7)	40.3 (7.6)	39.2 (7.9)	0.576	39.8 (7.8)	39.0 (8.0)	40.7 (7.2)	0.315
Education					0.598				0.789
Primary or secondary		46 (46.0)	41 (41.4)	39 (39.4)		45 (45.0)	39 (39.4)	42 (42.4)	
or higher		52 (52.0)	58 (58.6)	58 (58.6)		55 (55.0)	58 (58.6)	55 (55.6)	
Smoking, ever		42 (42.0)	45 (45.5)	42 (42.4)	0.866	47 (47.0)	39 (39.4)	43 (43.4)	0.556
Diagnosed diabetes		6 (6.0)	3 (3.03)	3 (3.03)	0.609	3 (3.0)	6 (6.1)	3 (3.03)	0.538
Heavy alcohol consumption		17 (17.0)	20 (20.2)	17 (17.7)	0.840	21 (21.0)	17 (17.2)	18 (18.2)	0.773
Hypertension		23 (23.0)	19 (19.2)	20 (20.2)	0.790	18 (18.0)	20 (20.2)	24 (24.24)	0.546
Obesity		43 (43.0)	45 (45.5)	53 (53.5)	0.298	41 (41.0)	46 (46.5)	54 (54.6)	0.157
History of stroke		58 (58.0)	56 (56.6)	32 (32.3)	**<0.001**	42 (42.0)	49 (49.5)	55 (55.6)	0.159
Antihypertensive medication^a^		5 (5.0)	6 (6.1)	3 (3.03)	0.625	2 (2.0)	6 (6.1)	6 (6.1)	0.296
Statin medication^a^		4 (4.0)	3 (3.03)	6 (6.1)	0.605	3 (3.0)	5 (5.1)	5 (5.1)	0.730
Antibiotic use^b^		10 (10.0)	11 (11.1)	23 (23.2)	**0.014**	11 (11.0)	13 (13.1)	20 (20.2)	0.160
Caries		36 (36.0)	41 (41.4)	41 (41.4)	0.666	35 (35.0)	49 (49.5)	34 (34.34)	**0.048**
Bleeding on probing (%)					0.400				0.328
	<30	29 (29.0)	22 (22.2)	32 (32.3)		35 (35.0)	24 (24.24)	24 (24.24)	
	30–50	53 (53.0)	49 (49.5)	45 (45.5)		49 (49.0)	48 (48.5)	50 (50.5)	
	>50	17 (17.0)	25 (25.3)	21 (21.2)		16 (16.0)	24 (24.24)	23 (23.2)	
					0.835				0.395
Number of all periodontal pockets (≥4 mm)	0–2	35 (35.0)	31 (31.3)	34 (34.3)		36 (36.0)	32 (32.3)	32 (32.3)	
	3–10	47 (47.0)	42 (42.4)	45 (45.5)		42 (42.0)	41 (41.4)	51 (51.5)	
	≥11	17 (17.0)	23 (23.2)	19 (19.2)		22 (22.0)	23 (23.2)	14 (14.1)	
Periodontal status					0.559				0.100
Healthy		23 (23.0)	16 (16.2)	25 (25.3)		29 (29.0)	18 (18.2)	17 (17.2)	
Gingivitis		50 (50.0)	56 (56.6)	51 (51.5)		53 (53.0)	54 (54.6)	50 (50.5)	
Periodontitis		26 (26.0)	24 (24.2)	21 (21.2)		17 (17.0)	24 (24.24)	30 (30.3)	

Data are expressed as *n* (%) unless otherwise indicated. Missing data: diagnosed diabetes *n* = 1, bleeding on probing *n* = 5, number of all periodontal pockets *n* = 5, periodontal status *n* = 6. Bold indicates *P* < 0.05.

a Current medication registered at recruitment time or within 6 mo.

b Prescribed antibiotic treatment within 6 mo before oral examination.

### Impact of Alpha Diversity and Oral Microbiome Composition on LPS

No significant correlation was observed between serum LPS levels and alpha diversity metrics (Shannon index, *R*^2^ = 0.072, *P* = 0.229; inverse Simpson index, *R*^2^ = 0.029, *P* = 0.625) (Fig. 1A). In contrast, a significant negative correlation was found between salivary LPS and Shannon (*R*^2^ = −0.124, *P* = 0.033) and inverse Simpson diversity (*R*^2^ = −0.162, *P* = 0.004, Fig. 1B). We found significant differences in Shannon (*P* = 0.047) and inverse Simpson (*P* = 0.009) diversity between the salivary LPS tertiles but not for serum (Fig. 1C, D). Both Shannon (*P* = 0.023) and inverse Simpson (*P* = 0.004) differed between salivary LPS tertiles 2 and 3. Linear regression analyses showed that serum LPS was positively associated with Shannon diversity index (*P* = 0.016), explaining 8.42% of the variance. Salivary LPS was negatively associated with inverse Simpson diversity (*P* = 0.017), while smoking and antibiotic use negatively affected both diversity indices. The model explained 9.0% of the variance in inverse Simpson diversity (Appendix Table 1).

**Figure 1.**
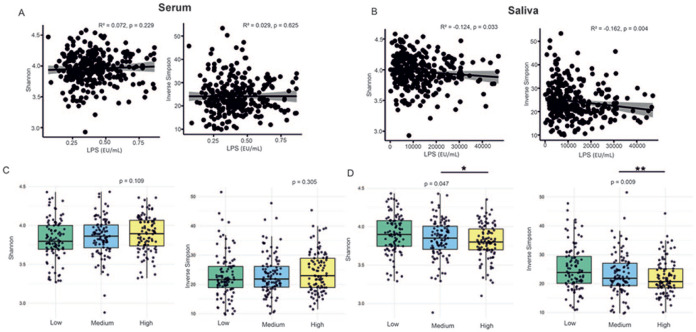
Associations between lipopolysaccharide (LPS) levels and microbial alpha diversity. Associations between (**A**) serum and (**B**) saliva LPS and alpha diversity (Shannon and inverse Simpson diversity). Comparison of Shannon and inverse Simpson diversity across (**C**) serum and (**D**) salivary LPS tertiles.

PERMANOVA showed that the microbiome composition did not significantly differ across the serum LPS tertiles (*F* = 0.80, *R*^2^ = 0.005, *P* = 0.675) (Fig. 2A). In contrast, the microbiome composition differed significantly between the salivary LPS tertiles (*F* = 3.60, *R*^2^ = 0.024, *P* = 0.001) (Fig. 2B). The differences were highly significant between all tertiles.

**Figure 2.**
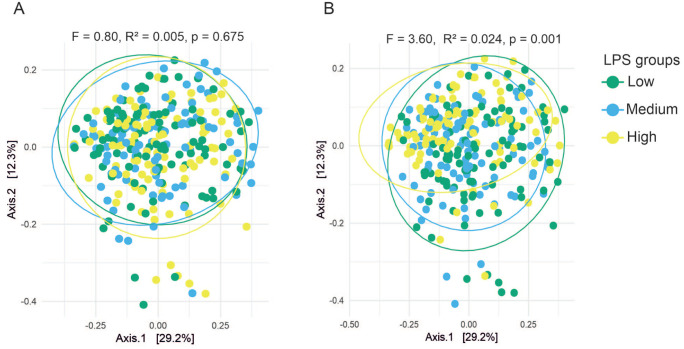
Principal coordinates analysis (PCoA) plots illustrate beta diversity (dissimilarity between microbial communities) using the Bray–Curtis dissimilarity at the species level for (**A**) serum and (**B**) salivary lipopolysaccharide (LPS) tertiles. Ellipses represent the 95% confidence intervals for each group.

### Taxonomic Composition and Differential Abundance

The oral microbiota was dominated by the phyla *Bacilliota*, *Actinomycetota*, *Pseudomonadota*, *Bacteroidota*, and *Fusobacteriota*. At the genus level, *Streptococcus*, *Prevotella*, *Neisseria*, *Veillonella*, and *Haemophilus* were dominant. We observed differences in abundance at the phylum and genus levels for both serum and salivary LPS tertiles (Fig. 3A, B). Venn diagrams show shared and unique core genera and species across the serum and salivary LPS tertiles (Fig. 3C, D). Among 89 genera found, 59 (66.3%) genera were shared across all serum LPS tertiles, whereas 7 (7.9%), 7 (7.9%), and 4 (4.5%) were unique for tertiles 1, 2, and 3, respectively. Among the 89 genera, 60 (67.4%) were shared between salivary tertiles, whereas 11 (12.4%), 2 (2.2%), and 7 (7.9%) were unique for tertiles 1, 2, and 3, respectively. *Cepunavirus (Streptococcus phage CP-7)*, *Leuconostoc*, and *Moraxella* were unique genera in both serum and saliva tertile 1, whereas *Staphylococcus* was unique in tertile 3 of both samples. Among the 276 identified species, 205 (74.3%) were shared across all serum LPS tertiles and 200 (74.6%) between the salivary LPS tertiles (Appendix Fig. 1).

**Figure 3.**
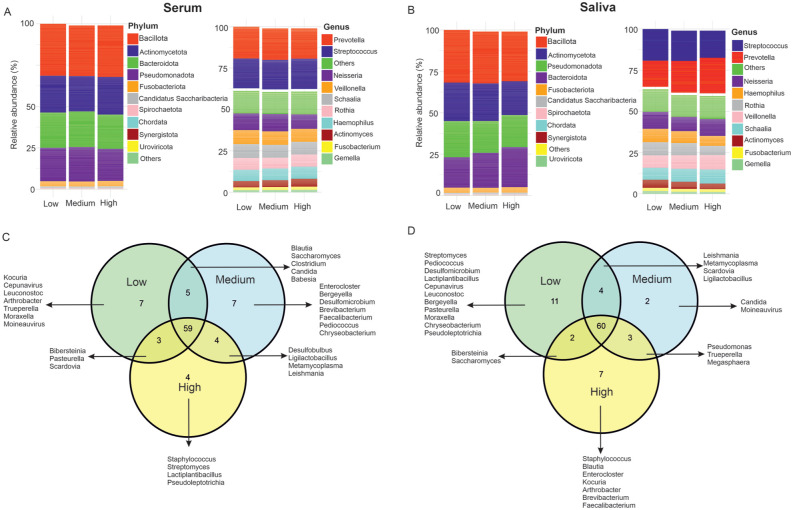
Microbial community composition in the saliva metagenome across lipopolysaccharide (LPS) tertiles. Stacked bar charts show the mean relative abundance of microbial taxa at the phylum and genus levels across different LPS tertiles for (**A**) serum and (**B**) saliva. Venn diagram depicting the shared and unique core genera across different (**C**) serum and (**D**) saliva LPS tertiles. Overlapping regions indicate genera shared between tertiles, while nonoverlapping areas represent unique core genera specific to each LPS tertile.

LEfSe analysis revealed that many taxa were differentially enriched between the serum and salivary LPS tertiles (Appendix Table 2). When comparing serum LPS tertiles, *Prevotella histicola* was significantly enriched in tertile 1, *Porphyromonas* species (including *P. gingivalis* and *P. endodontalis*) in tertile 2, and *Rothia dentocariosa* and *Fusobacterium nucleatum* in tertile 3. Salivary LPS tertiles showed even more significant differences. Genera *Streptococcus* (including 5 species), *Haemophilus* (1), *Rothia* (2), *Gemella* (2), and *Actinomyces* (3) were enriched in tertile 1. In contrast, taxa enriched in the LPS tertile 3 included the phylum *Bacteroidota* and genera *Prevotella* (including 3 species), *Veillonella* (1 species), *Schaalia* (1 species), *Leptotrichia* (1 species), and *Selenomonas* (2 species).

### Gram-Negative Bacteria and LPS

Among gram-negative species, no significant correlation was observed between alpha diversity metrics and serum or salivary LPS levels (Appendix Fig. 2A, B) or tertiles (Appendix Fig. 2C, D). Microbiome composition did not differ significantly between the serum LPS tertiles (*P* = 0.437), whereas the composition differed between salivary LPS tertiles (*F* = 3.72, *R*^2^ = 0.025, *P* = 0.001). *Bacteroidota* (especially genus *Prevotella*) increased, while *Bacillota* (especially *Streptococcus*) decreased with increasing salivary LPS tertiles (Appendix Fig. 2E, F).

### Oral Microbial Species as Predictors of LPS Levels

Multiple linear regression analysis was conducted to examine the relationship between bacterial genera or species and serum or salivary LPS levels. The model explained little variance in serum LPS levels (*F* = 1.022, *P* = 0.429) with no statistically significant core genera. In contrast, the model for salivary LPS was significant (*F* = 5.673, *P* < 0.001) and explained 19.3% of the variance. *Prevotella* (*P* < 0.001), *Neisseria* (*P* < 0.001), *Leptotrichia* (*P* < 0.001), and *Porphyromonas* (*P* = 0.020) showed significant positive associations with salivary LPS, whereas *Fusobacterium* (*P* = 0.041) showed a negative association (Appendix Table 3).

Further species-level analysis was performed based on the significant genera from the initial model, which explained 17.2% of the variance in salivary LPS levels (*P* < 0.001). *P. gingivalis* (*P* = 0.005), *L. wadei* (*P* < 0.001), and *Prevotella* sp. E13_17 (*P* < 0.001) were positively associated with salivary LPS, whereas *F. nucleatum* (*P* < 0.001) showed a negative association (Appendix Table 3).

### Metabolic Functional Shifts Associated with High LPS Levels

A total of 1,016 metabolic pathways were significantly associated with salivary LPS levels but not with serum LPS levels (Appendix Table 4). Metabolites associated with high salivary LPS levels were predominantly products of pathways related to biosynthesis of nucleosides, amino acids, and cofactors and electron carriers (Fig. 4A).

**Figure 4.**
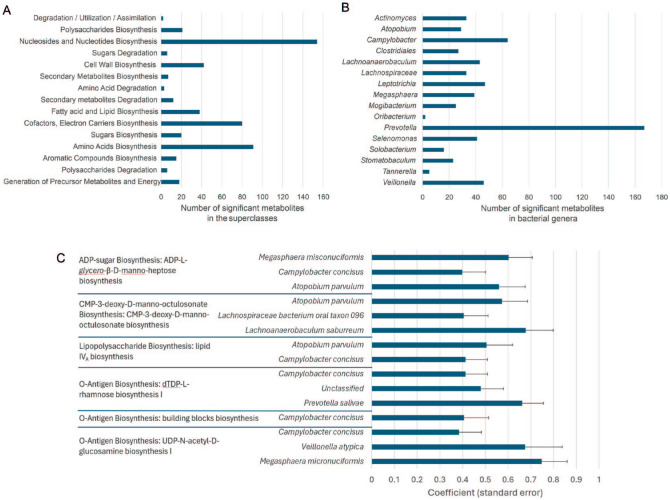
Functional pathway abundances differed significantly across saliva lipopolysaccharide (LPS) levels. (**A**) Number of significant metabolites grouped by metabolic super classes, (**B**) bacterial genera associated with the significant metabolites, and (**C**) specific metabolic pathways related to LPS biosynthesis, showing significant associations with high LPS levels. Key contributing taxa are indicated, with coefficients reflecting the strength of the association. Metabolic pathway profiles were generated using HUMAnN 3. Associations with saliva LPS levels were determined using MaAsLin 2.

At the taxonomic level, these metabolites were most frequently linked to members of the genera *Prevotella*, followed by *Campylobacter*, *Leptotrichia*, and *Veillonella* (Fig. 4B). Specific metabolic pathways involved in the biosynthesis of LPS, lipid A, and O-antigen were also significantly associated with high salivary LPS levels (*P* < 0.05) (Fig. 4C).

## Discussion

The present work shows that 33% to 34% of the oral genera and 25% to 26% of the species were associated with LPS levels in both specimens. Serum LPS activity was positively associated with richness and evenness of oral microbiome species, especially *P. gingivalis*, *P. endodontalis*, *R. dentocariosa*, and *F. nucleatum*. Still, the core genera explained only about 4% of the serum LPS variance, and none of them reached statistical significance. Salivary LPS activity was negatively associated with oral microbiome diversity, and the core microbiome composition differed between the LPS tertiles. A model composed of the core genera, *Prevotella*, *Neisseria*, *Leptotrichia*, *Porphyromonas*, and *Fusobacterium*, explained 19% of the salivary LPS variance. In addition, high salivary LPS levels were associated with functional pathways indicative of active microbial metabolism and proliferation within the oral microbiome. Oral microbiota–derived LPS activity in saliva demonstrated a reduction in oral microbiome variety and enrichment of obligate or facultative anaerobic species (i.e., typical signs of dysbiosis).

LPS and endotoxemia derived from the dysbiotic oral microbiome are strong stimulators of both local and systemic inflammatory and immunological responses (Pussinen et al. 2022). However, the oral microbiome composition or functionality associated with saliva or circulating LPS activity has not been investigated. In the present study, high serum LPS activity was related to high microbial diversity in saliva, but this was mainly characterized by gram-positive genera such as *Staphylococcus*, *Streptomyces*, and *Lactiplantibacillus*. In contrast, the analysis performed among gram-negative genera alone did not show significant associations. Furthermore, serum LPS activity was not associated with any metabolic profile of the oral microbiome. Despite weak relationships observed with serum LPS activity, our study does not exclude the hypothesis that serum LPS levels are associated with the composition of the oral microbiome.

In the present population, serum LPS activity was not significantly associated with periodontal status, but the serum LPS/lipoteichoic acid (LTA) ratio increased with increasing periodontitis severity (Leskelä et al. 2024). Severe periodontitis is characterized by reduced species richness and loss of biodiversity (Kumar 2017), whereas the present analyses revealed increased diversity with high serum LPS activity. It is possible that the subgingival plaque microbiome is more strongly associated with serum LPS activity than the saliva microbiome, but this has been explored in only 1 earlier study using targeted microbial analyses (Liljestrand et al. 2017). In that study, salivary LPS activity had a significant positive association with a cluster of subgingival gram-negative species, but no association with serum LPS activity was observed (Liljestrand et al. 2017), supporting our results. The oral microbiome may be primarily associated with serum LPS via the oral–gut axis (Kitamoto and Kamada 2022). Although people swallow 1.5 × 10^12^ oral bacteria daily, it has been estimated that more than 99.9% of the bacteria cannot survive the acidic environment of the stomach. However, some oral species, such as *Prevotella*, can pass through the stomach barrier and invade the terminal ileum and colon (Mukherjee et al. 2025). Thus, it is plausible that numerous bacterial cells, metabolites, or virulence factors derived from the oral cavity reach the gut, consequently contributing to dysbiosis, which may lead to increased gut permeability and endotoxemia (Mukherjee et al. 2025).

High salivary LPS was associated with decreased microbial diversity, suggesting that the microbiome is enriched by gram-negative species. Indeed, the microbiome of participants with high salivary LPS was enriched with gram-negative genera, such as *Campylobacter*, *Leptotrichia*, *Selenomonas*, and *Veillonella*, but especially *Prevotella*. This is aligned with our earlier results showing that salivary LPS activity is associated especially with plaque phyla *Pseudomonatota*, *Fusobacteriota*, and *Bacteroidota* (Kopra et al. 2021), whereas no association with the phylum *Spirochaetota* was observed. The outer membrane of *Spirochaetota* expresses several lipoproteins and glycolipids, such as LPS, but several *Treponem*a do not possess LPS or its chemical properties deviate from those described for classic gram-negative LPS (Schultz et al. 1998). The *Prevotella* genus, including species such as *P. histicola*, *P. intermedia*, *P. jejuni*, and *P. melaninogenica*, explained the highest proportion of salivary LPS variability (7.8%). These species are frequent colonizers of the oral cavity, with high proportions especially in saliva (Könönen and Gursoy 2022). Microbial communities and the host have a bilateral relationship, in which dysbiosis alters the immune responses resulting in inflammation, which further disrupts microbial homeostasis (Kilian et al. 2016). This also enhances the production of several virulence factors, such as LPS, leading to improved survival and increased pathogenicity (Curtis et al. 2020). LPS of *Prevotella* induces the release of proinflammatory cytokines from phagocytic cells, thus contributing to tissue destruction and bone resorption in periodontitis. The biological activity of LPS correlates with number, length, and saturation degree of its lipid A acyl chains. Longer acyl chains lead generally to stronger immune responses due to stronger hydrophobic interactions with immune cell receptors, such as toll-like receptor 4 accessory protein MD-2: hexa-acylated lipid A (e.g., from *Escherichia coli*) elicit strong responses, while penta-acylated (e.g., from *Prevotella*) or tetra-acylated forms (e.g., from *P. gingivalis*) trigger weaker or altered immune activation (Park et al. 2009; Sharma et al. 2022). However, *P. gingivalis* lipid A, for example, has a heterogeneous mixture of acyl chains containing 4 to 5 fatty acids (Reife et al. 2006). Thus, the composition of lipid A and its specific LPS activity vary between species and even between strains.

The predicted metabolic pathways associated with high salivary LPS levels were most commonly features of *Prevotella* species. Several enriched pathways reflected heightened biosynthetic activity, including nucleoside, amino acid, cofactor, and cell wall biosynthesis. These pathways are integral to microbial proliferation and consequently to the production of inflammatory molecules, potentially indicating a proinflammatory oral microenvironment. Although several bacterial fatty acids are involved lipid storage processes, their main location is the hydrophobic portion of the bacterial membrane (Cronan and Thomas 2009). The identified fatty acid biosynthesis pathways may also be involved in the biosynthesis of LPS because of their deviating paths to provide specific fatty acids, which are components of LPS lipid A, such as 3-hydroxy and short-chain fatty acids (Raetz and Whitfield, 2002). Other enriched pathways in high salivary LPS included those associated with the biosynthesis of O-antigen and KDO (3-deoxy-D-manno-octulosonic acid) in the inner core region of LPS, suggesting enhanced proliferation and virulence.

The use of any systemic antibiotics was registered up to 6 mo prior to recruitment. Antibiotic use was negatively associated with the diversity of the saliva microbiome. In earlier publications describing adult and adolescence populations, the postantibiotic recovery of the oral microbiome has been reported to last from 3 to 8 mo (Raju et al. 2020; Kopra et al. 2021). Similarly, several antibiotics prescribed during the preceding year were related to decreased serum and salivary LPS activity, whereas the effect was short-term and could be seen only until 1 mo (Kopra et al. 2021). Based on this information, we excluded from the present work participants who had used antibiotics within the preceding month.

It is important to note that the assay used is not specific to any bacterial species or LPS type but determines the activity derived from a mixture of LPS molecules in the sample. The biological activity of LPS depends on its structure; thus, the results are correlated with LPS concentration to only a limited extent (Munford 2016). However, the biological effects of LPS are determined by its activity, that is, the host response, which is a clinically relevant measure. Importantly, LPS activity assays are highly sensitive to environmental variables, such as the composition of different sample matrices, which cannot be directly compared with each other. The results need to be interpreted within these limitations, which emphasize the importance of further methodological validation in the future.

In conclusion, *Prevotella*, *Neisseria*, *Leptotrichia*, *Porphyromonas*, and *Fusobacterium* genera were the strongest determinants of salivary LPS activity, whereas no significant associations with serum LPS activity were observed. Oral microbiota–derived LPS activity in saliva was associated with highly active microbial metabolism characterized by proliferation, biosynthesis, and virulence. The observed taxonomic and functional signatures may enhance both local and systemic inflammation. Although the determination of salivary LPS activity may not be utilizable in clinical practice, it is a highly relevant measure in clinical research.

## Author Contributions

M. Manzoor, contributed to data acquisition, analysis, and interpretation, drafted the manuscript; J. Putaala, S. Paju, contributed to conception and design, data acquisition, analysis, and interpretation, critically revised the manuscript; S. Zaric, J. Leskelä, contributed to data acquisition, analysis, and interpretation, critically revised the manuscript; A Dong, contributed to data acquisition, critically revised the manuscript; E. Könönen, contributed to data acquisition and interpretation, critically revised the manuscript; L. Lahti, contributed to data interpretation, critically revised the manuscript; P. J. Pussinen, contributed to conception and design, data acquisition, analysis, and interpretation, drafted the manuscript. All authors gave final approval and agree to be accountable for all aspects of the work.

## Supplemental Material

sj-docx-1-jdr-10.1177_00220345251370995 – Supplemental material for Oral Microbial Determinants of Saliva and Serum Lipopolysaccharide Activity Supplemental material, sj-docx-1-jdr-10.1177_00220345251370995 for Oral Microbial Determinants of Saliva and Serum Lipopolysaccharide Activity by M. Manzoor, J. Putaala, S. Zaric, J. Leskelä, A. Dong, E. Könönen, L. Lahti, S. Paju and P. J. Pussinen in Journal of Dental Research
