# Supplementary material for: Oral Microbial Determinants of Saliva and Serum Lipopolysaccharide Activity
Source: J Dent Res. 2025 Oct 18;105(5):578–86. doi: 10.1177/00220345251370995 (PMC13068149; doi:10.1177/00220345251370995)
Supplement: Supplementary file 1 [file sj-docx-1-jdr-10.1177_00220345251370995.docx]

**Supplementary Data**

**Oral Microbial Determinants of Saliva and Serum Lipopolysaccharide Activity**

**M. Manzoor^1^, J. Putaala^2^, S. Zaric^3^, J. Leskelä^1^,** **A. Dong^3^, E. Könönen^4^, L. Lahti^5^, S. Paju^1^, P. J. Pussinen^1, 6^**

^1^ Department of Oral and Maxillofacial Diseases, University of Helsinki, Helsinki, Finland

^2^ Neurology, Helsinki University Hospital and University of Helsinki, Helsinki, Finland

^3^ Centre for Host-Microbiome Interactions, Faculty of Dentistry, Oral & Craniofacial Sciences, King’s College London, UK

^4^ Institute of Dentistry, University of Turku, Turku, Finland

^5^ Department of Computing, University of Turku, Turku, Finland

^6^ School of Medicine, Institute of Dentistry, University of Eastern Finland, Kuopio, Finland

**Corresponding authors:**

M Manzoor, Department of Oral and Maxillofacial Diseases, University of Helsinki, PO Box 63, Helsinki, 00014, Finland. email: muhammed.allippara@helsinki.fi

P. J. Pussinen, Department of Oral and Maxillofacial Diseases, University of Helsinki, PO Box 63, Helsinki, 00014, Finland and School of Medicine, Institute of Dentistry, University of Eastern Finland, Kuopio, Finland. email: pirkko.pussinen@helsinki.fi

**Supplementary Methods**

**Study Subjects and Recruitment**

At the time of recruitment, all participants underwent a thorough structured interview, and a detailed clinical history was obtained from medical records, including information on diabetes, hypertension, heavy alcohol consumption, abdominal obesity, and current or former tobacco smoking. Post-secondary or tertiary education was classified as a high level of education. Additionally, antimicrobial (antibiotic) medication, antihypertensive medication, and statin use were also recorded.

**Clinical Oral Examination**

The number of sites with probing pocket depth (PPD) of 4–5 mm, ≥6 mm, and bleeding on probing (BOP) was recorded from six sites per tooth. Regular dental check-ups, bifurcations, mobile teeth, cracked teeth, mucosal findings and the number of missing teeth were also recorded. Panoramic tomographs were obtained from all participants.

**Sample Collection and Metagenomic Sequencing**

The procedures for saliva sample collection, processing, DNA extraction, and sequencing have been previously described (Manzoor, 2024 a, b Manzoor, 2025). In brief, stimulated saliva samples were collected before the oral examination and transferred to RNase/DNase-free tubes, and 500 μl aliquots were mixed with an equal volume of lysis buffer in MN type B bead tubes (Macherey-Nagel) before storage at -80°C. DNA was extracted from using ChemagicTM 360 instrument (PerkinElmer) followed by metagenomic sequencing using an Illumina NovaSeq 6000 (San Diego, CA, USA). Sequence data preprocessing was conducted using FastQC (v. 0.11.9), MultiQC (v. 1.9), and Trimmomatic (v. 0.39). Kneaddata (v0.10.0) was used to remove human-associated sequences. High-quality, filtered reads were assigned taxonomy using Kraken2 (v2.1.2) (Wood et al. 2019), with species-level abundance refined by Bracken (v2.7.0).

**Statistical Analysis**

Summary statistics for numerical variables and counts and percentages for categorical variables were computed. ANOVA/Kruskal-Wallis (continuous) and χ^2^/Fisher’s exact (categorical) tests were performed.

We stored the sequence data and metadata in *Phyloseq* (v. 1.42.0). Before analysis, we filtered for taxa with >999 abundances, set prevalence (10%) and detection (0.01%) thresholds, and rarefied the data. We computed alpha diversity metrics (Shannon and Inverse Simpson indices), removed outliers from Serum LPS, and explored the relationship with LPS using scatter plots and Spearman correlations. We built a linear regression model to predict alpha diversity using LPS after adjusting for age, sex, smoking history, and recent antibiotic use. We performed Kruskal-Wallis tests for alpha diversity metrics across the LPS groups.

Permutational multivariate ANOVA (PERMANOVA) tests used the Bray-Curtis distance to assess differences based on LPS levels, both with 999 permutations adjusted for age, sex, smoking history, and recent antibiotic use. Post-hoc tests, including Tukey's Honest Significant Difference (HSD) test, were performed to identify pairwise differences. A principal coordinate analysis (PCoA) plot was used to visualize the beta diversity. We generated a stacked bar plot to visualize the relative abundance of the top taxa across different LPS levels and created a Venn diagram to identify core and unique taxa at the genus and species levels for each LPS group.

We performed Linear Discriminant Analysis Effect Size (LEfSe) analysis (Segata et al. 2011) at different taxonomic levels using LPS groups, with log10 transformation, CPM normalization, and Linear Discriminant Analysis (LDA) score > 3, applying Kruskal-Wallis and Wilcoxon tests (P < 0.05) for significance between LPS groups. Multiple linear regression models were fitted to identify the top gram-negative bacterial genera and significant species that predicted saliva and serum LPS levels, using microbial abundance data as independent variables and LPS levels as dependent variables.

Supplementary Sources

Manzoor M, Leskelä J, Pietiäinen M, Martinez-Majander N, Könönen E, Niiranen T, Lahti L, Sinisalo J, Putaala J, Pussinen PJ, Paju S. Shotgun metagenomic analysis of the oral microbiome in gingivitis: a nested case-control study. J Oral Microbiol. 2024 Mar 22;16(1):2330867. doi: 10.1080/20002297.2024.2330867. PMID: 38528961; PMCID: PMC10962305.

Manzoor M, Leskelä J, Pietiäinen M, Martinez-Majander N, Könönen E, Sinisalo J, Putaala J, Pussinen PJ, Paju S. Oral microbiome dysbiosis in cryptogenic ischemic stroke patients with high-risk patent foramen ovale. Sci. Rep. 2025; 15(1):11535. doi: 10.1038/s41598-025-95728-x

Manzoor M, Leskelä J, Pietiäinen M, Martinez-Majander N, Ylikotila P, Könönen E, Niiranen T, Lahti L, Sinisalo J, Putaala J, Pussinen PJ, Paju S. Multikingdom oral microbiome interactions in early-onset cryptogenic ischemic stroke. ISME Commun. 2024 Jun 20;4(1):ycae088. doi: 10.1093/ismeco/ycae088. PMID: 38988699; PMCID: PMC11235082.

Segata N, Izard J, Waldron L, Gevers D, Miropolsky L, Garrett WS, Huttenhower C. Metagenomic biomarker discovery and explanation. Genome Biol. 2011 Jun 24;12(6):R60. doi: 10.1186/gb-2011-12-6-r60.

Wood DE, Lu J, Langmead B. Improved metagenomic analysis with Kraken 2. Genome Biol. 2019 Nov 28;20(1):257. doi: 10.1186/s13059-019-1891-0. PMID: 31779668; PMCID: PMC6883579.

**Supplementary Figure 1.** Core microbiome analysis at the species level. Venn diagram depicting the shared and unique core species across different serum and saliva LPS groups. Core species were defined based on a detection threshold of 0.1% relative abundance. Overlapping regions indicate species shared between groups, while non-overlapping areas represent unique core species specific to each LPS group.

**Supplementary Figure 2. Gram-negative bacterial diversity and composition across serum and saliva LPS levels.** (A) Scatterplots showing correlations between serum LPS and alpha diversity. (B) Box plots of Shannon and Inverse Simpson diversity across serum LPS groups. (C) PCoA plots illustrating beta diversity (Bray-Curtis) and genus-level composition across serum LPS tertiles. (D) Scatterplots showing correlations between saliva LPS and alpha diversity. (E) Box plots of Shannon and Inverse Simpson diversity across saliva LPS groups and (F) PCoA plots illustrating beta diversity (Bray-Curtis) and genus-level composition across saliva LPS groups.

**Supplementary Table 1.** Linear regression analysis of saliva metagenome predictors on alpha diversity (Shannon and Inverse Simpson) in relation to saliva and serum LPS.

|  | **Shannon** | | | | **Inverse Simpson** | | | |
| --- | --- | --- | --- | --- | --- | --- | --- | --- |
| **Predictor** | **Estimate** | **Std. Error** | **t-value** | **p-value** | **Estimate** | **Std. Error** | **t-value** | **p-value** |
| **(Intercept)** | 3.89 | 0.0865 | 44.96 | **<0.0001** | 21.17 | 2.55 | 8.29 | **<0.0001** |
| **Age** | 0.0019 | 0.0019 | 0.985 | 0.326 | 0.0956 | 0.055 | 1.72 | 0.086 |
| **Saliva LPS** | -2.2e-06 | 1.4e-06 | -1.559 | 0.120 | -1.0e-04 | 4.3e-05 | -2.39 | **0.017** |
| **Serum LPS** | 0.1455 | 0.0601 | 2.419 | **0.016** | 3.299 | 1.775 | 1.85 | 0.064 |
| **Gender (Male)** | 0.0467 | 0.0294 | 1.590 | 0.113 | 1.658 | 0.8664 | 1.91 | 0.057 |
| **Smoking (Ever = yes)** | -0.0803 | 0.0285 | -2.822 | **0.005** | -2.532 | 0.8397 | -3.01 | **0.003** |
| **Antibiotic use (6M)** | -0.1213 | 0.0405 | -2.995 | **0.003** | -2.937 | 1.195 | -2.45 | **0.015** |

Model Summary: Shannon Diversity: Residual Std. Error: 0.243, Multiple R²: 0.0842, Adjusted R²: 0.0653, F-statistic: 4.459 (p = 0.00025). Inverse Simpson Diversity: Residual Std. Error: 7.172, Multiple R²: 0.0900, Adjusted R²: 0.0713 and F-statistic: 4.798 (p = 0.00011)

**Supplementary Table 2. List of differentially abundant microbial taxa across different taxonomic levels identified using Lefse in saliva and serum LPS groups. Only taxa with an LDA score >3 are shown.**

| **Serum** | | | | |
| --- | --- | --- | --- | --- |
| **Genus** | **Species** | **Enriched tertile** | **LDA Score** | **Adjusted p-value** |
| *Lancefieldella* |  | Low | 3.190 | 0.021 |
|  | *Lancefieldella parvula* | Low | 3.185 | 0.021 |
|  | *Porphyromonas endodontalis* | Low | 3.090 | 0.049 |
|  | *Porphyromonas gingivalis* | Low | 3.047 | 0.039 |
|  | *Prevotella histicola* | Low | 3.841 | 0.037 |
| *Porphyromonas* |  | Medium | 3.359 | 0.005 |
|  | *Porphyromonas gingivalis* | Medium | 3.357 | 0.008 |
|  | *Porphyromonas endodontalis* | Medium | 3.106 | 0.004 |
|  | *Fusobacterium nucleatum* | High | 3.149 | 0.026 |
|  | *Rothia dentocariosa* | High | 3.454 | 0.010 |
| **Saliva LPS** | | | | |
| **Phylum / Genus** | **Species** | **Enriched tertile** | **LDA Score** | **adjusted p-value** |
| *Actinomyces* |  | Low-medium | 4.161 | 0.012 |
|  | *Actinomyces oris* | Low | 3.560 | 0.031 |
|  | *Actinomyces sp. oral taxon 171* | Low | 3.073 | 0.002 |
|  | *Actinomyces sp. oral taxon 414* | Low | 3.641 | 0.019 |
| *Actinomycetota* |  | Low-medium | 4.435 | 0.022 |
|  | *Fusobacterium nucleatum* | Low | 3.304 | 0.010 |
| *Gemella* |  | Low | 3.713 | <0.001 |
|  | *Gemella haemolysans* | Low | 3.280 | 0.005 |
|  | *Gemella sanguinis* | Low | 3.511 | 0.013 |
| *Granulicatella* |  | Low | 3.270 | 0.023 |
| *Haemophilus* |  | Low | 4.311 | 0.002 |
|  | *Haemophilus parainfluenzae* | Low | 4.281 | 0.003 |
| *Rothia* |  | Low-medium | 4.354 | <0.001 |
|  | *Rothia aeria* | Low | 3.429 | 0.001 |
|  | *Rothia dentocariosa* | Low | 3.783 | 0.002 |
|  | *Rothia mucilaginosa* | Low | 4.167 | <0.001 |
| *Streptococcus* |  | Low | 4.412 | 0.004 |
|  | *Streptococcus australis* | Low | 3.106 | 0.025 |
|  | *Streptococcus mitis* | Low | 3.853 | 0.032 |
|  | *Streptococcus pneumoniae* | Low | 3.267 | 0.009 |
|  | *Streptococcus sanguinis* | Low | 3.217 | 0.026 |
|  | *Streptococcus sp. A12* | Low | 3.119 | 0.046 |
|  | *Veillonella parvula* | Low | 3.315 | 0.027 |
| *Actinomycetota* |  | Medium | 4.360 | 0.013 |
| *Actinomyces* |  | Medium | 3.938 | 0.001 |
|  | *Actinomyces oris* | Medium | 3.440 | 0.002 |
|  | *Actinomyces sp. HMT 175* | Medium | 3.048 | 0.008 |
|  | *Actinomyces sp. oral taxon 169* | Medium | 3.195 | 0.004 |
|  | *Actinomyces sp. oral taxon 414* | Medium | 3.200 | 0.012 |
| *Olsenella* |  | Medium | 3.210 | 0.004 |
|  | *Olsenella sp. oral taxon 807* | Medium | 3.122 | 0.003 |
| *Prevotella* |  | Medium | 4.543 | 0.012 |
|  | *Prevotella jejuni* | Medium | 4.242 | 0.017 |
|  | *Prevotella melaninogenica* | Medium | 4.020 | 0.048 |
| *Rothia* |  | Medium | 4.223 | <0.001 |
|  | *Rothia dentocariosa* | Medium | 3.591 | 0.001 |
|  | *Rothia mucilaginosa* | Medium | 4.217 | 0.005 |
|  | *Veillonella atypica* | Medium | 3.715 | 0.039 |
| *Bacteroidota* |  | High | 4.801 | <0.001 |
| *Prevotella* |  | High | 4.782 | <0.001 |
|  | *Prevotella histicola* | Medium-high | 4.153 | 0.003 |
|  | *Prevotella intermedia* | High | 3.318 | 0.001 |
|  | *Prevotella jejuni* | Medium-high | 4.422 | <0.001 |
|  | *Prevotella melaninogenica* | Medium-high | 4.250 | 0.001 |
| *Campylobacter* |  | High | 3.154 | 0.013 |
|  | *Campylobacter concisus* | High | 3.270 | 0.018 |
| *Lancefieldella* |  | High | 3.299 | 0.020 |
|  | *Lancefieldella parvula* | High | 3.298 | 0.020 |
| *Leptotrichia* |  | High | 3.706 | 0.012 |
|  | *Leptotrichia wadei* | High | 3.586 | 0.005 |
| *Mogibacterium* |  | High | 3.043 | <0.001 |
| *Schaalia* |  | High | 4.166 | 0.015 |
|  | *Schaalia odontolytica* | High | 4.171 | 0.015 |
| *Selenomonas* |  | High | 3.769 | 0.003 |
|  | *Selenomonas sp. oral taxon 136* | High | 3.333 | <0.001 |
|  | *Selenomonas sp. oral taxon 478* | High | 3.463 | <0.001 |
| *Veillonella* |  | High | 4.1384 | 0.027 |
|  | *Veillonella atypica* | Medium-high | 4.0167 | <0.001 |

**Supplementary Table 3.** Associations of oral microbiome and with serum and saliva LPS levels.

|  | Serum | | | Saliva | | |
| --- | --- | --- | --- | --- | --- | --- |
| **Model 1** | | | | | | |
|  | **β** | t | *P* value | **β** | t | *P* value |
| (Constant) |  | 5.663 | <0.001 |  | 1.179 | 0.239 |
| *Prevotella* | -0.051 | -.619 | 0.536 | 0.315 | 4.197 | **<0.001** |
| *Neisseria* | -0.071 | -.954 | 0.341 | 0.263 | 3.826 | **<0.001** |
| *Leptotrichia* | 0.039 | .619 | 0.536 | 0.210 | 3.639 | **<0.001** |
| *Porphyromonas* | -0.137 | -1.578 | 0.116 | 0.187 | 2.349 | **0.020** |
| *Tannerella* | 0.175 | 1.802 | 0.073 | 0.117 | 1.305 | 0.193 |
| *Aggregatibacter* | -0.006 | -.083 | 0.934 | 0.034 | .551 | 0.582 |
| *Kingella* | 0.036 | .567 | 0.571 | 0.029 | .498 | 0.619 |
| *Campylobacter* | 0.068 | 1.015 | 0.311 | 0.012 | .188 | 0.851 |
| *Haemophilus* | 0.038 | .486 | 0.627 | -0.051 | -.725 | 0.469 |
| *Lautropia* | -0.059 | -.900 | 0.369 | -0.064 | -1.059 | 0.291 |
| *Fusobacterium* | 0.029 | .361 | 0.718 | -0.151 | -2.053 | **0.041** |
| *Treponema* | -0.020 | -.190 | 0 .849 | -0.159 | -1.682 | 0.094 |
| **Model 2** | | | | | | |
| (Constant) | | | |  | 6.933 | <0.001 |
| *Prevotella* spp. E13_17 | | | | 0.230 | 4.152 | **<0.001** |
| *Leptotrichia wadei* | | | | 0.222 | 3.973 | **<0.001** |
| *Porphyromonas gingivalis* | | | | 0.165 | 2.854 | **0.005** |
| *Neisseria weixii* | | | | 0.068 | 1.252 | 0.212 |
| *Fusobacterium nucleatum* | | | | -0.210 | -3.572 | **<0.001** |

Model 1: Serum LPS: Residual Std. Error: 0.2381, Multiple R²: 0.041, Adjusted R²: .001, F-statistic: 1.022 and *P* = 0.429. Saliva LPS: Residual Std. Error: 9029, Multiple R²: 0.193, Adjusted R²: 0.159, F-statistic: 5.673 and *P* = <0.001.

Model 2: Residual Std. Error: 0.903, Multiple R²: 0.172, Adjusted R²: .158, F-statistic: 12.12 and p = <0.001

**Supplymentary Table S4.** Metabolic pathways associated with saliva LPS activity.

| **Feature** | **Genus** | **Species** | **Coefficient** | **qval** |
| --- | --- | --- | --- | --- |
| COBALSYN.PWY..superpathway.of.adenosylcobalamin.  salvage.from.cobinamide.I | Prevotella | salivae | 0,723 | 0,000 |
| GLUCUROCAT.PWY..superpathway.of..beta..  D lucuronosides.degradation | Prevotella | salivae | 0,738 | 0,000 |
| NONMEVIPP.PWY..methylerythritol.  phosphate.pathway.I | Prevotella | salivae | 0,737 | 0,000 |
| PWY.3841..folate.transformations.II..plants | Prevotella | salivae | 0,744 | 0,000 |
| PWY.8178..pentose.phosphate.pathway..non.oxidative.  branch..II | Prevotella | salivae | 0,698 | 0,000 |
| PWY.6507..4.deoxy.L.threo.hex.4.enopyranuronate.  degradation | Prevotella | salivae | 0,723 | 0,000 |
| PWY.5686..UMP.biosynthesis.I | Leptotrichia | wadei | 0,673 | 0,000 |
| PWY.7790..UMP.biosynthesis.II | Leptotrichia | wadei | 0,673 | 0,000 |
| PWY.7791..UMP.biosynthesis.III | Leptotrichia | wadei | 0,673 | 0,000 |
| PWY.7851..coenzyme.A.biosynthesis.II..eukaryotic | Prevotella | salivae | 0,706 | 0,000 |
| RIBOSYN2.PWY..flavin.biosynthesis.I..bacteria.and.plants | Prevotella | salivae | 0,789 | 0,000 |
| PWY.6700..queuosine.biosynthesis.I..de.novo | Prevotella | salivae | 0,914 | 0,000 |
| ANAG.glycolysis.PWY.glycolysis.III..from lucose | Prevotella | salivae | 0,664 | 0,000 |
| PWY.5686..UMP.biosynthesis.I | Prevotella | oulorum | 0,344 | 0,000 |
| PWY.7221 uanosine.ribonucleotides.de.novo.biosynthesis | Prevotella | oulorum | 0,716 | 0,000 |
| PWY.7790..UMP.biosynthesis.II | Prevotella | oulorum | 0,344 | 0,000 |
| PWY.7791..UMP.biosynthesis.III | Prevotella | oulorum | 0,344 | 0,000 |
| TRNA.CHARGING.PWY..tRNA.charging | Prevotella | salivae | 0,817 | 0,000 |
| PWY.6151..S.adenosyl.L.methionine.salvage.I | Prevotella | salivae | 1,022 | 0,000 |
| ARO.PWY..chorismate.biosynthesis.I | Prevotella | salivae | 0,660 | 0,000 |
| PWY.5686..UMP.biosynthesis.I | Prevotella | salivae | 0,740 | 0,000 |
| PWY.7790..UMP.biosynthesis.II | Prevotella | salivae | 0,740 | 0,000 |
| PWY.7791..UMP.biosynthesis.III | Prevotella | salivae | 0,740 | 0,000 |
| PWY.6121..5.aminoimidazole.ribonucleotide.biosynthesis.I | Stomatobaculum | longum | 0,441 | 0,000 |
| PWY.7221 uanosine.ribonucleotides.de.novo.biosynthesis | Prevotella | salivae | 0,876 | 0,000 |
| PWY.1269..CMP.3.deoxy.D.manno.octulosonate.biosynthesis | Prevotella | salivae | 0,662 | 0,000 |
| VALSYN.PWY..L.valine.biosynthesis | Stomatobaculum | longum | 0,588 | 0,000 |
| PWY.621..sucrose.degradation.III..sucrose.invertase | Prevotella | salivae | 0,677 | 0,000 |
| GALACTUROCAT.PWY..D alacturonate.degradation.I | Prevotella | salivae | 0,715 | 0,000 |
| PWY.1042.glycolysis.IV | Prevotella | oulorum | 0,377 | 0,000 |
| PWY.6386..UDP.N.acetylmuramoyl.pentapeptide.  biosynthesis.II..lysine.containing | Prevotella | salivae | 0,638 | 0,000 |
| ARGSYNBSUB.PWY..L.arginine.biosynthesis.II..acetyl.cycle | Lachnoanaerobaculum | saburreum | 0,512 | 0,000 |
| PWY.6147..6.hydroxymethyl.dihydropterin.  diphosphate.biosynthesis.I | Prevotella | salivae | 0,723 | 0,000 |
| PWY.7242..D.fructuronate.degradation | Prevotella | salivae | 0,670 | 0,000 |
| ARGSYN.PWY..L.arginine.biosynthesis.I..via.L.ornithine | Lachnoanaerobaculum | saburreum | 0,417 | 0,000 |
| PWY.6124..inosine.5..phosphate.biosynthesis.II | Prevotella | salivae | 0,943 | 0,000 |
| PWY.6700..queuosine.biosynthesis.I..de.novo | Lachnoanaerobaculum | saburreum | 0,681 | 0,000 |
| SER LYSYN.PWY..superpathway.of.L.serine.and  lycine.biosynthesis.I | Prevotella | salivae | 0,685 | 0,000 |
| GLYCOGENSYNTH.PWY lycogen.biosynthesis.  I..from.ADP.D lucose | Solobacterium | moorei | 0,719 | 0,000 |
| PWY.5667..CDP.diacylglycerol.biosynthesis.I | Prevotella | salivae | 0,739 | 0,000 |
| PWY.724..superpathway.of.L.lysine..L.threonine.and  .L.methionine.biosynthesis.II | Prevotella | salivae | 0,635 | 0,000 |
| PWY0.1319..CDP.diacylglycerol.biosynthesis.II | Prevotella | salivae | 0,739 | 0,000 |
| COA.PWY..coenzyme.A.biosynthesis.I..prokaryotic | Lachnoanaerobaculum | saburreum | 0,586 | 0,000 |
| PWY.5973..cis.vaccenate.biosynthesis | Prevotella | salivae | 1,007 | 0,000 |
| COA.PWY..coenzyme.A.biosynthesis.I..prokaryotic | Prevotella | salivae | 0,709 | 0,000 |
| PWY.6121..5.aminoimidazole.ribonucleotide.biosynthesis.I | Lachnoanaerobaculum | saburreum | 0,536 | 0,000 |
| PWY.6385..peptidoglycan.biosynthesis.III..mycobacteria | Prevotella | salivae | 0,702 | 0,000 |
| PWY.I9..L.cysteine.biosynthesis.VI..from.L.methionine | Leptotrichia | wadei | 0,714 | 0,000 |
| PWY.7953..UDP.N.acetylmuramoyl.pentapeptide.  biosynthesis.III..meso.diaminopimelate.containing | Prevotella | salivae | 0,987 | 0,000 |
| PWY.6121..5.aminoimidazole.ribonucleotide.biosynthesis.I | Solobacterium | moorei | 0,511 | 0,000 |
| PWY.6122..5.aminoimidazole.ribonucleotide.biosynthesis.II | Stomatobaculum | longum | 0,456 | 0,000 |
| PWY.6277..superpathway.of.5.aminoimidazole  .ribonucleotide.biosynthesis | Stomatobaculum | longum | 0,456 | 0,000 |
| PWY.6123..inosine.5..phosphate.biosynthesis.I | Prevotella | salivae | 0,918 | 0,000 |
| PWY0.1586..peptidoglycan.maturation..meso.  diaminopimelate.containing | Prevotella | salivae | 0,698 | 0,000 |
| PWY.6163..chorismate.biosynthesis.from.3.dehydroquinate | Stomatobaculum | longum | 0,519 | 0,000 |
| PWY.5188..tetrapyrrole.biosynthesis.I..from lutamate | Lachnoanaerobaculum | saburreum | 0,437 | 0,000 |
| PWY.5667..CDP.diacylglycerol.biosynthesis.I | Mogibacterium | diversum | 0,373 | 0,000 |
| PWY.6122..5.aminoimidazole.ribonucleotide.biosynthesis.II | Mogibacterium | diversum | 0,553 | 0,000 |
| PWY.6277..superpathway.of.5.aminoimidazole  .ribonucleotide.biosynthesis | Mogibacterium | diversum | 0,553 | 0,000 |
| PWY0.1319..CDP.diacylglycerol.biosynthesis.II | Mogibacterium | diversum | 0,373 | 0,000 |
| PWY0.1296..purine.ribonucleosides.degradation | Solobacterium | moorei | 0,524 | 0,000 |
| PWY0.1297..superpathway.of.purine.  deoxyribonucleosides.degradation | Lachnoanaerobaculum | saburreum | 0,394 | 0,000 |
| PWY.5989..stearate.biosynthesis.II..bacteria.and.plants | Prevotella | salivae | 0,911 | 0,000 |
| PWY0.1241..ADP.L lycero..beta..D.manno  .heptose.biosynthesis | Megasphaera | micronuciformis | 0,747 | 0,000 |
| PWY0.862...5Z..dodecenoate.biosynthesis.I | Prevotella | salivae | 0,867 | 0,000 |
| CALVIN.PWY..Calvin.Benson.Bassham.cycle | Prevotella | salivae | 0,618 | 0,000 |
| PWY.5103..L.isoleucine.biosynthesis.III | Prevotella | salivae | 0,945 | 0,000 |
| PWY.5097..L.lysine.biosynthesis.VI | Prevotella | salivae | 0,741 | 0,000 |
| PWY.6163..chorismate.biosynthesis.from.3.dehydroquinate | Prevotella | salivae | 0,600 | 0,000 |
| ILEUSYN.PWY..L.isoleucine.biosynthesis.I..from.threonine | Prevotella | salivae | 0,894 | 0,000 |
| PWY.7664..oleate.biosynthesis.IV..anaerobic | Prevotella | salivae | 0,864 | 0,000 |
| PWY.6282..palmitoleate.biosynthesis.I..from..  5Z..dodec.5.enoate | Prevotella | salivae | 0,881 | 0,000 |
| FASYN.ELONG.PWY..fatty.acid.elongation....saturated | Prevotella | salivae | 0,861 | 0,000 |
| PWY.2942..L.lysine.biosynthesis.III | Prevotella | salivae | 0,769 | 0,000 |
| ARO.PWY..chorismate.biosynthesis.I | Lachnoanaerobaculum | saburreum | 0,489 | 0,000 |
| PWY.7851..coenzyme.A.biosynthesis.II..eukaryotic | Lachnoanaerobaculum | saburreum | 0,532 | 0,000 |
| PWY.2942..L.lysine.biosynthesis.III | Leptotrichia | wadei | 0,648 | 0,000 |
| DTDPRHAMSYN.PWY..dTDP..beta.  .L.rhamnose.biosynthesis | Prevotella | salivae | 0,673 | 0,000 |
| PWY.6122..5.aminoimidazole.  ribonucleotide.biosynthesis.II | Lachnoanaerobaculum | saburreum | 0,449 | 0,000 |
| PWY.6277..superpathway.of.5.aminoimidazole  .ribonucleotide.biosynthesis | Lachnoanaerobaculum | saburreum | 0,449 | 0,000 |
| PWY.6703..preQ0.biosynthesis | Prevotella | salivae | 0,845 | 0,000 |
| DTDPRHAMSYN.PWY..dTDP..beta..  L.rhamnose.biosynthesis | Prevotella | oulorum | 0,278 | 0,000 |
| GLUCUROCAT.PWY..superpathway.of..beta  \..D lucuronosides.degradation |  |  | 0,551 | 0,000 |
| PWY0.1296..purine.ribonucleosides.degradation | Lachnoanaerobaculum | saburreum | 0,608 | 0,000 |
| PWY.6609..adenine.and.adenosine.salvage.III | Prevotella | salivae | 0,922 | 0,000 |
| HSERMETANA.PWY..L.methionine.biosynthesis.III | Prevotella | salivae | 0,730 | 0,000 |
| ARO.PWY..chorismate.biosynthesis.I | Stomatobaculum | longum | 0,450 | 0,000 |
| PWY.6700..queuosine.biosynthesis.I..de.novo | Stomatobaculum | longum | 0,509 | 0,000 |
| UNINTEGRATED | Stomatobaculum | longum | 0,795 | 0,000 |
| PWY.6151..S.adenosyl.L.methionine.salvage.I | Leptotrichia | wadei | 0,772 | 0,000 |
| PWY.6122..5.aminoimidazole.ribonucleotide.  biosynthesis.II | Solobacterium | moorei | 0,538 | 0,000 |
| PWY.6277..superpathway.of.5.aminoimidazole.  ribonucleotide.biosynthesis | Solobacterium | moorei | 0,538 | 0,000 |
| PWY66.429..fatty.acid.biosynthesis.initiation..mitochondria | Lachnoanaerobaculum | saburreum | 0,628 | 0,000 |
| GALACTUROCAT.PWY..D alacturonate.degradation.I |  |  | 0,628 | 0,000 |
| UNINTEGRATED | Mogibacterium | diversum | 0,566 | 0,000 |
| PWY.6507..4.deoxy.L.threo.hex.4.enopyranuronate.degradation |  |  | 0,602 | 0,000 |
| PWY66.429..fatty.acid.biosynthesis.initiation..mitochondria | Mogibacterium | diversum | 0,524 | 0,000 |
| PEPTIDOGLYCANSYN.PWY..peptidoglycan.  biosynthesis.I..meso.diaminopimelate.containing | Prevotella | salivae | 0,911 | 0,000 |
| ANAG.glycolysis.PWY.glycolysis.III..from lucose | Lachnoanaerobaculum | saburreum | 0,499 | 0,000 |
| PWY.7111..pyruvate.fermentation.to.isobutanol..engineered | Lachnoanaerobaculum | saburreum | 0,573 | 0,000 |
| GLYCOGENSYNTH.PWY lycogen.  biosynthesis.I..from.ADP.D lucose | Lachnoanaerobaculum | saburreum | 0,574 | 0,000 |
| PWY.6609..adenine.and.adenosine.salvage.III | Lachnoanaerobaculum | saburreum | 0,538 | 0,000 |
| PWY.2942..L.lysine.biosynthesis.III | Megasphaera | micronuciformis | 0,749 | 0,000 |
| PWY.5686..UMP.biosynthesis.I | Stomatobaculum | longum | 0,415 | 0,000 |
| PWY.7790..UMP.biosynthesis.II | Stomatobaculum | longum | 0,415 | 0,000 |
| PWY.7791..UMP.biosynthesis.III | Stomatobaculum | longum | 0,415 | 0,000 |
| PWY.7851..coenzyme.A.biosynthesis.II..eukaryotic | Megasphaera | micronuciformis | 0,755 | 0,000 |
| PWY.5695..inosine.5..phosphate.degradation | Prevotella | salivae | 0,885 | 0,000 |
| PWY.7238..sucrose.biosynthesis.II | Lachnoanaerobaculum | saburreum | 0,634 | 0,000 |
| DTDPRHAMSYN.PWY..dTDP..beta..L.rhamnose  .biosynthesis | Stomatobaculum | longum | 0,715 | 0,000 |
| COA.PWY.1..superpathway.of.coenzyme.A  .biosynthesis.III..mammals | Lachnoanaerobaculum | saburreum | 0,507 | 0,000 |
| GLYCOGENSYNTH.PWY lycogen.biosynthesis.I.  .from.ADP.D lucose | Stomatobaculum | longum | 0,432 | 0,000 |
| PWY.6897..thiamine.diphosphate.salvage.II | Megasphaera | micronuciformis | 0,581 | 0,000 |
| TRNA.CHARGING.PWY..tRNA.charging | Mogibacterium | diversum | 0,306 | 0,000 |
| GLUTORN.PWY..L.ornithine.biosynthesis.I | Lachnoanaerobaculum | saburreum | 0,482 | 0,000 |
| COA.PWY.1..superpathway.of.coenzyme.A.  biosynthesis.III..mammals | Megasphaera | micronuciformis | 0,748 | 0,000 |
| PWY.5097..L.lysine.biosynthesis.VI | Megasphaera | micronuciformis | 0,732 | 0,000 |
| PWY.6387..UDP.N.acetylmuramoyl.pentapeptide.  biosynthesis.I..meso.diaminopimelate.containing | Prevotella | salivae | 0,892 | 0,000 |
| PWY.7977..L.methionine.biosynthesis.IV | Prevotella | salivae | 0,665 | 0,000 |
| PWY.6387..UDP.N.acetylmuramoyl.pentapeptide.  biosynthesis.I..meso.diaminopimelate.containing | Stomatobaculum | longum | 0,366 | 0,000 |
| PWY.5097..L.lysine.biosynthesis.VI | Leptotrichia | wadei | 0,586 | 0,000 |
| PWY.5097..L.lysine.biosynthesis.VI | Lachnoanaerobaculum | saburreum | 0,314 | 0,000 |
| PWY.6121..5.aminoimidazole.ribonucleotide.biosynthesis.I | Mogibacterium | diversum | 0,454 | 0,000 |
| PWY.6121..5.aminoimidazole.ribonucleotide.biosynthesis.I | Megasphaera | micronuciformis | 0,647 | 0,000 |
| PWY.7197..pyrimidine.deoxyribonucleotide.phosphorylation | Mogibacterium | diversum | 0,626 | 0,000 |
| PWY.1042.glycolysis.IV | Prevotella | salivae | 0,728 | 0,000 |
| PWY.6386..UDP.N.acetylmuramoyl.pentapeptide.  biosynthesis.II..lysine.containing | Stomatobaculum | longum | 0,370 | 0,000 |
| PWY.6969..TCA.cycle.V..2.oxoglutarate.synthase..unclassified |  |  | 0,546 | 0,000 |
| PWY.6163..chorismate.biosynthesis.from.3.dehydroquinate | Lachnoanaerobaculum | saburreum | 0,464 | 0,000 |
| RIBOSYN2.PWY..flavin.biosynthesis.I..bacteria.and.plants | Megasphaera | micronuciformis | 0,605 | 0,000 |
| PWY.7238..sucrose.biosynthesis.II | Stomatobaculum | longum | 0,618 | 0,000 |
| THRESYN.PWY..superpathway.of.L.threonine.biosynthesis | Megasphaera | micronuciformis | 0,585 | 0,000 |
| PWY.6386..UDP.N.acetylmuramoyl.pentapeptid  e.biosynthesis.II..lysine.containing | Megasphaera | micronuciformis | 0,602 | 0,000 |
| ILEUSYN.PWY..L.isoleucine.biosynthesis.I..from.threonine | Lachnoanaerobaculum | saburreum | 0,507 | 0,000 |
| PWY.6163..chorismate.biosynthesis.from.3.dehydroquinate | Mogibacterium | diversum | 0,369 | 0,000 |
| PWY.6609..adenine.and.adenosine.salvage.III | Solobacterium | moorei | 0,507 | 0,000 |
| PWY.2942..L.lysine.biosynthesis.III | Lachnoanaerobaculum | saburreum | 0,294 | 0,000 |
| PWY.7977..L.methionine.biosynthesis.IV | Leptotrichia | wadei | 0,723 | 0,000 |
| PWY.724..superpathway.of.L.lysine..L.  threonine.and.L.methionine.biosynthesis.II | Megasphaera | micronuciformis | 0,560 | 0,000 |
| PWY.7221 uanosine.ribonucleotides.  de.novo.biosynthesis | Mogibacterium | diversum | 0,580 | 0,000 |
| PEPTIDOGLYCANSYN.PWY..peptidoglycan.  biosynthesis.I..meso.diaminopimelate.containing | Stomatobaculum | longum | 0,363 | 0,000 |
| PWY.8131..5..deoxyadenosine.degradation.II.unclassified |  |  | 0,470 | 0,000 |
| UNINTEGRATED | Lachnoanaerobaculum | saburreum | 0,631 | 0,000 |
| VALSYN.PWY..L.valine.biosynthesis | Prevotella | salivae | 0,777 | 0,000 |
| PWY.5103..L.isoleucine.biosynthesis.III | Lachnoanaerobaculum | saburreum | 0,514 | 0,000 |
| NONMEVIPP.PWY..methylerythritol.  phosphate.pathway.I | Stomatobaculum | longum | 0,289 | 0,000 |
| PWY.5667..CDP.diacylglycerol.biosynthesis.I | Megasphaera | micronuciformis | 0,598 | 0,000 |
| PWY0.1319..CDP.diacylglycerol.biosynthesis.II | Megasphaera | micronuciformis | 0,598 | 0,000 |
| PWY.6823..molybdopterin.biosynthesis | Megasphaera | micronuciformis | 0,806 | 0,000 |
| PWY.7111..pyruvate.fermentation.to.isobutanol.. | Megasphaera | micronuciformis | 0,703 | 0,000 |
| PWY.2941..L.lysine.biosynthesis.II | Leptotrichia | wadei | 0,558 | 0,000 |
| BRANCHED.CHAIN.AA.SYN.PWY..superpathway  .of.branched.chain.amino.acid.biosynthesis | Lachnoanaerobaculum | saburreum | 0,558 | 0,000 |
| PWY.6700..queuosine.biosynthesis.I..de.novo | Megasphaera | micronuciformis | 0,620 | 0,000 |
| PWY.3841..folate.transformations.II..plants | Mogibacterium | diversum | 0,381 | 0,000 |
| PWY.6122..5.aminoimidazole.ribonucleotide.  biosynthesis.II | Prevotella | salivae | 0,762 | 0,000 |
| PWY.6277..superpathway.of.5.aminoimidazole  .ribonucleotide.biosynthesis | Prevotella | salivae | 0,762 | 0,000 |
| PWY66.429..fatty.acid.biosynthesis.initiation..mitochondria | Leptotrichia | wadei | 0,614 | 0,000 |
| GLUTORN.PWY..L.ornithine.biosynthesis.I | Leptotrichia | wadei | 0,690 | 0,000 |
| NONMEVIPP.PWY..methylerythritol.phosphate.pathway.I | Mogibacterium | diversum | 0,434 | 0,000 |
| ARGSYN.PWY..L.arginine.biosynthesis.I..via.L.ornithine | Leptotrichia | wadei | 0,472 | 0,000 |
| VALSYN.PWY..L.valine.biosynthesis | Megasphaera | micronuciformis | 0,689 | 0,000 |
| PWY.6609..adenine.and.adenosine.salvage.III | Stomatobaculum | longum | 0,446 | 0,000 |
| COMPLETE.ARO.PWY..superpathway.of.aromatic.  \amino.acid.biosynthesis | Lachnoanaerobaculum | saburreum | 0,428 | 0,000 |
| PWY.6609..adenine.and.adenosine.salvage.III | Leptotrichia | wadei | 0,662 | 0,000 |
| PWY.6609..adenine.and.adenosine.salvage.III | Megasphaera | micronuciformis | 0,707 | 0,000 |
| PWY.6151..S.adenosyl.L.methionine.salvage.I | Lachnoanaerobaculum | saburreum | 0,446 | 0,000 |
| PWY.6151..S.adenosyl.L.methionine.salvage.I | Stomatobaculum | longum | 0,426 | 0,000 |
| PEPTIDOGLYCANSYN.PWY..peptidoglycan  .biosynthesis.I..meso.diaminopimelate.containing | Megasphaera | micronuciformis | 0,700 | 0,000 |
| PWY.7371..1.4.dihydroxy.6.naphthoate.biosynthesis.II |  |  | 0,601 | 0,000 |
| PANTOSYN.PWY..superpathway.of.coenzyme.  A.biosynthesis.I..bacteria. |  |  | 0,073 | 0,000 |
| UDPNAGSYN.PWY..UDP.N.acetyl.D lucosamine.  biosynthesis.I | Megasphaera | micronuciformis | 0,603 | 0,000 |
| P41.PWY..pyruvate.fermentation.to.acetate.and..S..lactate.I | Solobacterium | moorei | 0,488 | 0,000 |
| PWY.5100..pyruvate.fermentation.to.acetate.and.lactate.II | Solobacterium | moorei | 0,488 | 0,000 |
| PWY.6385..peptidoglycan.biosynthesis.III..mycobacteria | Megasphaera | micronuciformis | 0,539 | 0,000 |
| COA.PWY..coenzyme.A.biosynthesis.I..prokaryotic | Leptotrichia | wadei | 0,577 | 0,000 |
| PWY.7371..1.4.dihydroxy.6.naphthoate.biosynthesis.II |  |  | 0,522 | 0,000 |
| PWY.1042.glycolysis.IV | Lachnoanaerobaculum | saburreum | 0,551 | 0,000 |
| PWY.6703..preQ0.biosynthesis | Megasphaera | micronuciformis | 0,602 | 0,000 |
| PWY0.1586..peptidoglycan.maturation..meso.  diaminopimelate.containing | Megasphaera | micronuciformis | 0,735 | 0,000 |
| PWY.7221 uanosine.ribonucleotides.de.novo.biosynthesis | Solobacterium | moorei | 0,478 | 0,000 |
| PWY.6122..5.aminoimidazole.ribonucleotide.biosynthesis.II | Megasphaera | micronuciformis | 0,596 | 0,000 |
| PWY.6277..superpathway.of.5.aminoimidazole.  ribonucleotide.biosynthesis | Megasphaera | micronuciformis | 0,596 | 0,000 |
| PWY.702..L.methionine.biosynthesis.II | Leptotrichia | wadei | 0,686 | 0,000 |
| PWY.6387..UDP.N.acetylmuramoyl.pentapeptide.biosynthesis  .I..meso.diaminopimelate.containing | Megasphaera | micronuciformis | 0,714 | 0,000 |
| PWY.7953..UDP.N.acetylmuramoyl.pentapeptide.biosynthesis.  III..meso.diaminopimelate.containing | Megasphaera | micronuciformis | 0,525 | 0,000 |
| DTDPRHAMSYN.PWY..dTDP..beta..L.rhamnose.biosynthesis | Leptotrichia | wadei | 0,550 | 0,000 |
| PWY.5695..inosine.5..phosphate.degradation | Megasphaera | micronuciformis | 0,688 | 0,000 |
| PWY.7221 uanosine.ribonucleotides.de.novo.biosynthesis | Leptotrichia | wadei | 0,615 | 0,000 |
| GLYCOGENSYNTH.PWY lycogen.biosynthesis.I..  from.ADP.D lucose | Atopobium | parvulum | 0,638 | 0,000 |
| PWY.7992..superpathway.of.menaquinol.8.  \biosynthesis.III |  |  | 0,561 | 0,000 |
| PWY.5686..UMP.biosynthesis.I | Megasphaera | micronuciformis | 0,634 | 0,000 |
| PWY.7790..UMP.biosynthesis.II | Megasphaera | micronuciformis | 0,634 | 0,000 |
| PWY.7791..UMP.biosynthesis.III | Megasphaera | micronuciformis | 0,634 | 0,000 |
| PWY.7357..thiamine.phosphate.formation.from  .pyrithiamine.and.oxythiamine..yeast | Megasphaera | micronuciformis | 0,557 | 0,000 |
| PWY.7238..sucrose.biosynthesis.II | Leptotrichia | wadei | 0,653 | 0,000 |
| PWY.6123..inosine.5..phosphate.biosynthesis.I | Leptotrichia | wadei | 0,644 | 0,000 |
| PWY.6385..peptidoglycan.biosynthesis.III..mycobacteria | Leptotrichia | wadei | 0,537 | 0,000 |
| P41.PWY..pyruvate.fermentation.to.acetate.and..S..lactate.I | Lachnoanaerobaculum | saburreum | 0,459 | 0,000 |
| PWY.5100..pyruvate.fermentation.to.acetate.and.lactate.II | Lachnoanaerobaculum | saburreum | 0,459 | 0,000 |
| PWY.6163..chorismate.biosynthesis.from.3.dehydroquinate | Megasphaera | micronuciformis | 0,622 | 0,000 |
| PWY.5686..UMP.biosynthesis.I |  |  | 0,053 | 0,000 |
| ASPASN.PWY..superpathway.of.L.aspartate.  and.L.asparagine.biosynthesis | Prevotella | salivae | 0,544 | 0,000 |
| VALSYN.PWY..L.valine.biosynthesis | Lachnoanaerobaculum | saburreum | 0,548 | 0,000 |
| PWY.5667..CDP.diacylglycerol.biosynthesis.I | Leptotrichia | wadei | 0,627 | 0,000 |
| PWY0.1319..CDP.diacylglycerol.biosynthesis.II | Leptotrichia | wadei | 0,627 | 0,000 |
| PWY.5941 lycogen.degradation.II | Lachnoanaerobaculum | saburreum | 0,439 | 0,000 |
| NONMEVIPP.PWY..methylerythritol.phosphate.pathway.I |  |  | 0,079 | 0,000 |
| PWY.6122..5.aminoimidazole.ribonucleotide.biosynthesis.II | Atopobium | parvulum | 0,662 | 0,000 |
| PWY.6277..superpathway.of.5.aminoimidazole.  \ribonucleotide.biosynthesis | Atopobium | parvulum | 0,662 | 0,000 |
| PWY.5973..cis.vaccenate.biosynthesis | Prevotella | oulorum | 0,602 | 0,000 |
| PWY.6147..6.hydroxymethyl.dihydropterin.diphosphate  .biosynthesis.I | Megasphaera | micronuciformis | 0,581 | 0,000 |
| DTDPRHAMSYN.PWY..dTDP..beta..L.rhamnose.  biosynthesis | Lachnoanaerobaculum | saburreum | 0,677 | 0,000 |
| PWY.7851..coenzyme.A.biosynthesis.II..eukaryotic | Solobacterium | moorei | 0,391 | 0,000 |
| ARGSYN.PWY..L.arginine.biosynthesis.I..via.L.  ornithine..unclassified |  |  | 0,459 | 0,000 |
| PWY.6386..UDP.N.acetylmuramoyl.pentapeptide.  biosynthesis.II..lysine.containing | Leptotrichia | wadei | 0,544 | 0,000 |
| PWY0.1296..purine.ribonucleosides.degradation | Megasphaera | micronuciformis | 0,644 | 0,000 |
| COA.PWY..coenzyme.A.biosynthesis.I..prokaryotic | Selenomonas | flueggei | 0,369 | 0,000 |
| PWY.7221 uanosine.ribonucleotides.de.novo.biosynthesis | Atopobium | parvulum | 0,605 | 0,000 |
| PWY.5667..CDP.diacylglycerol.biosynthesis.I | Stomatobaculum | longum | 0,383 | 0,000 |
| PWY0.1319..CDP.diacylglycerol.biosynthesis.II | Stomatobaculum | longum | 0,383 | 0,000 |
| PWY.6124..inosine.5..phosphate.biosynthesis.II | Leptotrichia | wadei | 0,668 | 0,000 |
| PWY.7242..D.fructuronate.degradation |  |  | 0,448 | 0,000 |
| UNINTEGRATED | Prevotella | salivae | 0,643 | 0,000 |
| X1CMET2.PWY..folate.transformations.III..E..coli | Leptotrichia | wadei | 0,365 | 0,000 |
| BRANCHED.CHAIN.AA.SYN.PWY..superpathway.  of.branched.chain.amino.acid.biosynthesis | Selenomonas | flueggei | 0,309 | 0,000 |
| UNINTEGRATED | Prevotella | oulorum | 0,602 | 0,000 |
| PWY.5667..CDP.diacylglycerol.biosynthesis.I | Campylobacter | concisus | 0,394 | 0,000 |
| PWY0.1319..CDP.diacylglycerol.biosynthesis.II | Campylobacter | concisus | 0,394 | 0,000 |
| SER LYSYN.PWY..superpathway.of.L.serine.and  lycine.biosynthesis.I | Selenomonas | flueggei | 0,362 | 0,000 |
| COA.PWY.1..superpathway.of.coenzyme.A.b  iosynthesis.III..mammals | Mogibacterium | diversum | 0,428 | 0,000 |
| UNINTEGRATED | Leptotrichia | wadei | 0,721 | 0,000 |
| PWY.7851..coenzyme.A.biosynthesis.II..eukaryotic | Mogibacterium | diversum | 0,442 | 0,000 |
| COA.PWY..coenzyme.A.biosynthesis.I..prokaryotic | Solobacterium | moorei | 0,490 | 0,000 |
| PWY.6803..phosphatidylcholine.acyl.editing | Campylobacter | concisus | 0,570 | 0,000 |
| PWY.6901..superpathway.of lucose.and.xylose.  degradation.unclassified |  |  | 0,394 | 0,000 |
| PWY.7953..UDP.N.acetylmuramoyl.pentapeptide.biosynthesis  .III..meso.diaminopimelate.containing | Leptotrichia | wadei | 0,587 | 0,000 |
| PWY.6387..UDP.N.acetylmuramoyl.pentapeptide.biosynthesis  .I..meso.diaminopimelate.containing | Mogibacterium | diversum | 0,304 | 0,000 |
| PWY.6386..UDP.N.acetylmuramoyl.pentapeptide  \.biosynthesis.II..lysine.containing | Mogibacterium | diversum | 0,302 | 0,000 |
| PWY.7992..superpathway.of.menaquinol.8.biosynthesis.III. |  |  | 0,407 | 0,000 |
| PWY.5695..inosine.5..phosphate.degradation | Atopobium | parvulum | 0,555 | 0,000 |
| PWY.5918..superpathway.of.heme.b.biosynthesis.  from lutamate.unclassified |  |  | 0,438 | 0,000 |
| NONOXIPENT.PWY..pentose.phosphate.pathway  ..non.oxidative.branch..I | Lachnoanaerobaculum | saburreum | 0,391 | 0,000 |
| COA.PWY..coenzyme.A.biosynthesis.I..prokaryotic | Megasphaera | micronuciformis | 0,675 | 0,000 |
| PWY.6549..L lutamine.biosynthesis.III |  |  | 0,544 | 0,000 |
| HISTSYN.PWY..L.histidine.biosynthesis | Leptotrichia | wadei | 0,540 | 0,000 |
| DTDPRHAMSYN.PWY..dTDP..beta..L.rhamnose.biosynthesis |  |  | 0,062 | 0,000 |
| PWY.6122..5.aminoimidazole.ribonucleotide.biosynthesis.II | Campylobacter | concisus | 0,599 | 0,000 |
| PWY.6277..superpathway.of.5.aminoimidazole  .ribonucleotide.biosynthesis | Campylobacter | concisus | 0,599 | 0,000 |
| UNINTEGRATED | Megasphaera | micronuciformis | 0,771 | 0,000 |
| HISDEG.PWY..L.histidine.degradation.I | Stomatobaculum | longum | 0,397 | 0,000 |
| ARGSYNBSUB.PWY..L.arginine.biosynthesis.II.  .acetyl.cycle..unclassified |  |  | 0,547 | 0,000 |
| PWY.6126..superpathway.of.adenosine.nucleotides.  de.novo.biosynthesis.II | Campylobacter | concisus | 0,395 | 0,000 |
| P124.PWY..Bifidobacterium.shunt | Atopobium | parvulum | 0,521 | 0,000 |
| TRNA.CHARGING.PWY..tRNA.charging | Leptotrichia | wadei | 0,510 | 0,000 |
| PWY.1042.glycolysis.IV | Clostridiales |  | 0,321 | 0,000 |
| NONMEVIPP.PWY..methylerythritol.phosphate.pathway.I | Prevotella | sp oral taxon 306 | 0,671 | 0,000 |
| CALVIN.PWY..Calvin.Benson.Bassham.cycle | Selenomonas | flueggei | 0,337 | 0,000 |
| PWY.7229..superpathway.of.adenosine.nucleotides.  de.novo.biosynthesis.I | Campylobacter | concisus | 0,400 | 0,000 |
| THISYNARA.PWY..superpathway.of.thiamine.  diphosphate.biosynthesis.III..eukaryotes | Prevotella | pallens | 0,541 | 0,000 |
| PWY.7456...beta...1.4..mannan.degradation |  |  | 0,509 | 0,000 |
| PWY.6151..S.adenosyl.L.methionine.salvage.I | Mogibacterium | diversum | 0,393 | 0,000 |
| HSERMETANA.PWY..L.methionine.biosynthesis.III | Leptotrichia | wadei | 0,687 | 0,000 |
| PWY.7790..UMP.biosynthesis.II |  |  | 0,054 | 0,000 |
| PWY.7791..UMP.biosynthesis.III |  |  | 0,054 | 0,000 |
| PWY.6608 uanosine.nucleotides.degradation.III | Megasphaera | micronuciformis | 0,670 | 0,000 |
| PWY.6823..molybdopterin.biosynthesis | Lachnoanaerobaculum | saburreum | 0,422 | 0,000 |
| THRESYN.PWY..superpathway.of.L.threonine.biosynthesis | Selenomonas | flueggei | 0,278 | 0,000 |
| PWY.7238..sucrose.biosynthesis.II | Solobacterium | moorei | 0,454 | 0,000 |
| PWY.6700..queuosine.biosynthesis.I..de.novo | Leptotrichia | wadei | 0,560 | 0,000 |
| ARGSYNBSUB.PWY..L.arginine.biosynthesis.II..acetyl.cycle | Leptotrichia | wadei | 0,552 | 0,000 |
| GALACT LUCUROCAT.PWY..superpathway.of  .hexuronide.and.hexuronate.degradation |  |  | 0,446 | 0,000 |
| PYRIDNUCSYN.PWY..NAD.de.novo.biosynthesis.I..from.aspartate | Prevotella | pallens | 0,512 | 0,000 |
| UNINTEGRATED | Selenomonas | noxia | 0,449 | 0,000 |
| PWY.7221 uanosine.ribonucleotides.de.novo.biosynthesis | Lachnoanaerobaculum | saburreum | 0,362 | 0,000 |
| RIBOSYN2.PWY..flavin.biosynthesis.I..bacteria.and.plants | Prevotella | pallens | 0,570 | 0,000 |
| PWY.7221 uanosine.ribonucleotides.de.novo.biosynthesis | Megasphaera | micronuciformis | 0,641 | 0,000 |
| COA.PWY.1..superpathway.of.coenzyme.A  .biosynthesis.III..mammals | Solobacterium | moorei | 0,177 | 0,000 |
| PWY.6151..S.adenosyl.L.methionine.salvage.I | Prevotella | pallens | 0,512 | 0,000 |
| PWY.6121..5.aminoimidazole.ribonucleotide.biosynthesis.I | Leptotrichia | wadei | 0,651 | 0,000 |
| PWY.8178..pentose.phosphate.pathway..non.oxidative.branch..II | Lachnoanaerobaculum | saburreum | 0,367 | 0,000 |
| PWY.5464..superpathway.of.cytosolic .glycolysis..plants..  .pyruvate.dehydrogenase.and.TCA.cycle |  |  | 0,439 | 0,000 |
| ALLANTOINDEG.PWY..superpathway.of  .allantoin.degradation.in.yeast |  |  | 0,485 | 0,000 |
| PWY.6163..chorismate.biosynthesis.from.3.dehydroquinate | Leptotrichia | wadei | 0,531 | 0,000 |
| PWY.2942..L.lysine.biosynthesis.III | Selenomonas | flueggei | 0,351 | 0,000 |
| PWY.7663 ondoate.biosynthesis..anaerobic | Prevotella | salivae | 0,768 | 0,000 |
| PWY.5005..biotin.biosynthesis.II | Veillonella | atypica | 0,673 | 0,000 |
| TRNA.CHARGING.PWY..tRNA.charging | Solobacterium | moorei | 0,231 | 0,000 |
| PWY.7663 ondoate.biosynthesis..anaerobic | Prevotella | oulorum | 0,526 | 0,000 |
| PWY.6122..5.aminoimidazole.ribonucleotide.biosynthesis.II | Leptotrichia | wadei | 0,682 | 0,000 |
| PWY.6277..superpathway.of.5.aminoimidazole.  ribonucleotide.biosynthesis | Leptotrichia | wadei | 0,682 | 0,000 |
| OANTIGEN.PWY..O.antigen.building.blocks.biosynthesis..E..coli | Atopobium | parvulum | 0,573 | 0,000 |
| PWY.6147..6.hydroxymethyl.dihydropterin.diphosphate.biosynthesis.I | Prevotella | pallens | 0,681 | 0,000 |
| PWY.5686..UMP.biosynthesis.I | Atopobium | parvulum | 0,492 | 0,000 |
| PWY.7790..UMP.biosynthesis.II | Atopobium | parvulum | 0,492 | 0,000 |
| PWY.7791..UMP.biosynthesis.III | Atopobium | parvulum | 0,492 | 0,000 |
| PWY.3841..folate.transformations.II..plants | Prevotella | pallens | 0,745 | 0,000 |
| COA.PWY..coenzyme.A.biosynthesis.I..prokaryotic | Mogibacterium | diversum | 0,452 | 0,000 |
| PEPTIDOGLYCANSYN.PWY..peptidoglycan.biosynthesis  .I..meso.diaminopimelate.containing | Mogibacterium | diversum | 0,255 | 0,000 |
| UNINTEGRATED | Solobacterium | moorei | 0,502 | 0,000 |
| PWY.7238..sucrose.biosynthesis.II | Atopobium | parvulum | 0,550 | 0,000 |
| PEPTIDOGLYCANSYN.PWY..peptidoglycan.biosynthesis.  I..meso.diaminopimelate.containing | Lachnoanaerobaculum | saburreum | 0,242 | 0,000 |
| PWY0.1296..purine.ribonucleosides.degradation | Clostridiales_Family_XIII |  | 0,412 | 0,000 |
| TRNA.CHARGING.PWY..tRNA.charging | Lachnoanaerobaculum | saburreum | 0,260 | 0,000 |
| PWY.6386..UDP.N.acetylmuramoyl.pentapeptide.biosynthesis.  II..lysine.containing | Clostridiales_Family_XIII |  | 0,385 | 0,000 |
| PWY.6163..chorismate.biosynthesis.from.3.dehydroquinate |  |  | 0,053 | 0,000 |
| PWY.2942..L.lysine.biosynthesis.III | Atopobium | parvulum | 0,474 | 0,000 |
| PWY.6387..UDP.N.acetylmuramoyl.pentapeptide.biosynthesis.  I..meso.diaminopimelate.containing | Clostridiales_Family_XIII |  | 0,392 | 0,000 |
| COMPLETE.ARO.PWY..superpathway.of.aromatic.amino.  acid.biosynthesis | Prevotella | pallens | 0,562 | 0,000 |
| PANTOSYN.PWY..superpathway.of.coenzyme.A.  biosynthesis.I..bacteria | Campylobacter | concisus | 0,401 | 0,000 |
| PWY.5097..L.lysine.biosynthesis.VI.unclassified |  |  | 0,269 | 0,000 |
| PWY.7238..sucrose.biosynthesis.II | Lachnospiraceae | bacterium oral taxon 96 | 0,506 | 0,000 |
| PWY.1042.glycolysis.IV | Selenomonas | flueggei | 0,251 | 0,000 |
| PWY.6386..UDP.N.acetylmuramoyl.pentapeptide.biosynthesis  .II..lysine.containing | Lachnoanaerobaculum | saburreum | 0,255 | 0,000 |
| PWY.6387..UDP.N.acetylmuramoyl.pentapeptide.biosynthesis  .I..meso.diaminopimelate.containing | Lachnoanaerobaculum | saburreum | 0,249 | 0,000 |
| PWY.7663 ondoate.biosynthesis..anaerobic..unclassified |  |  | 0,223 | 0,000 |
| COA.PWY.1..superpathway.of.coenzyme.A.biosynthesis.I  II..mammals | Campylobacter | concisus | 0,480 | 0,000 |
| PANTO.PWY..phosphopantothenate.biosynthesis.I | Campylobacter | concisus | 0,520 | 0,000 |
| PANTO.PWY..phosphopantothenate.biosynthesis.I |  |  | 0,074 | 0,000 |
| PWY.5973..cis.vaccenate.biosynthesis | Campylobacter | concisus | 0,561 | 0,000 |
| PWY.7371..1.4.dihydroxy.6.naphthoate.biosynthesis.II | Selenomonas | flueggei | 0,363 | 0,000 |
| PWY.6123..inosine.5..phosphate.biosynthesis.I | Prevotella | pallens | 0,701 | 0,000 |
| PWY.5667..CDP.diacylglycerol.biosynthesis.I | Prevotella | pallens | 0,760 | 0,000 |
| PWY0.1319..CDP.diacylglycerol.biosynthesis.II | Prevotella | pallens | 0,760 | 0,000 |
| PWY.7111..pyruvate.fermentation.to.isobutanol..engineered | Leptotrichia | wadei | 0,548 | 0,000 |
| PWY0.1296..purine.ribonucleosides.degradation | Leptotrichia | wadei | 0,603 | 0,000 |
| PWY.5686..UMP.biosynthesis.I | Mogibacterium | diversum | 0,314 | 0,000 |
| PWY.7790..UMP.biosynthesis.II | Mogibacterium | diversum | 0,314 | 0,000 |
| PWY.7791..UMP.biosynthesis.III | Mogibacterium | diversum | 0,314 | 0,000 |
| FASYN.ELONG.PWY..fatty.acid.elongation....saturated.unclassified |  |  | 0,482 | 0,000 |
| PWY.6628..superpathway.of.L.phenylalanine.biosynthesis.unclassified |  |  | 0,398 | 0,000 |
| PWY.7664..oleate.biosynthesis.IV..anaerobic..unclassified |  |  | 0,493 | 0,000 |
| PEPTIDOGLYCANSYN.PWY..peptidoglycan.biosynthesis.  I..meso.diaminopimelate.containing | Leptotrichia | wadei | 0,513 | 0,000 |
| PWY.7228..superpathway.of uanosine.nucleotides.de.novo.biosynthesis.I | Mogibacterium | diversum | 0,432 | 0,000 |
| PWY.6292..superpathway.of.L.cysteine.biosynthesis.  .mammalian..unclassified |  |  | 0,453 | 0,000 |
| PWY.5690..TCA.cycle.II..plants.and.fungi. |  |  | 0,460 | 0,000 |
| ILEUSYN.PWY..L.isoleucine.biosynthesis.I..from.threonine | Selenomonas | flueggei | 0,381 | 0,000 |
| PWY.5103..L.isoleucine.biosynthesis.III | Selenomonas | flueggei | 0,415 | 0,000 |
| PWY.7953..UDP.N.acetylmuramoyl.pentapeptide.biosynthesis.  III..meso.diaminopimelate.containing | Campylobacter | concisus | 0,311 | 0,000 |
| HISDEG.PWY..L.histidine.degradation.I | Prevotella | pallens | 0,631 | 0,000 |
| UDPNAGSYN.PWY..UDP.N.acetyl.D lucosamine.biosynthesis.I | Atopobium | parvulum | 0,560 | 0,000 |
| PWY.6387..UDP.N.acetylmuramoyl.pentapeptide.biosynthesis.I  ..meso.diaminopimelate.containing | Campylobacter | concisus | 0,389 | 0,000 |
| PWY.7851..coenzyme.A.biosynthesis.II..eukaryotic | Campylobacter | concisus | 0,483 | 0,000 |
| PWY.7198..pyrimidine.deoxyribonucleotides.de.novo.biosynthesis.IV | Actinomyces | graevenitzii | 0,569 | 0,000 |
| PWY.6700..queuosine.biosynthesis.I..de.novo | Mogibacterium | diversum | 0,407 | 0,000 |
| NONMEVIPP.PWY..methylerythritol.phosphate.pathway.I.unclassified |  |  | 0,276 | 0,000 |
| PPGPPMET.PWY..ppGpp.metabolism | Campylobacter | concisus | 0,316 | 0,000 |
| SER LYSYN.PWY..superpathway.of.L.serine.and lycine.biosynthesis.I | Prevotella | pallens | 0,732 | 0,000 |
| PWY.1269..CMP.3.deoxy.D.manno.octulosonate.biosynthesis.unclassified |  |  | 0,480 | 0,000 |
| PWY.6122..5.aminoimidazole.ribonucleotide.biosynthesis.II | Prevotella | pallens | 0,592 | 0,000 |
| PWY.6277..superpathway.of.5.aminoimidazole.ribonucleotide.biosynthesis | Prevotella | pallens | 0,592 | 0,000 |
| PWY.6282..palmitoleate.biosynthesis.I..from..5Z..dodec.5.enoate..unclassified |  |  | 0,454 | 0,000 |
| GLUTORN.PWY..L.ornithine.biosynthesis.I.unclassified |  |  | 0,497 | 0,000 |
| COA.PWY..coenzyme.A.biosynthesis.I..prokaryotic | Campylobacter | concisus | 0,492 | 0,000 |
| ARO.PWY..chorismate.biosynthesis.I.unclassified |  |  | 0,229 | 0,000 |
| PWY.7953..UDP.N.acetylmuramoyl.pentapeptide.biosynthesis  .III..meso.diaminopimelate.containing. |  |  | 0,059 | 0,000 |
| NONOXIPENT.PWY..pentose.phosphate.pathway..non.  oxidative.branch..I | Campylobacter | concisus | 0,480 | 0,000 |
| PWY.6121..5.aminoimidazole.ribonucleotide.biosynthesis.I | Atopobium | parvulum | 0,385 | 0,000 |
| PWY.6700..queuosine.biosynthesis.I..de.novo | Atopobium | parvulum | 0,478 | 0,000 |
| PWY.7111..pyruvate.fermentation.to.isobutanol..engineered | Selenomonas | flueggei | 0,410 | 0,000 |
| PWY.6387..UDP.N.acetylmuramoyl.pentapeptide.biosynthesis.I.  .meso.diaminopimelate.containing | Leptotrichia | wadei | 0,537 | 0,000 |
| COA.PWY.1..superpathway.of.coenzyme.A.biosynthesis.III..mammals | Veillonella | atypica | 0,771 | 0,000 |
| PWY.6385..peptidoglycan.biosynthesis.III..mycobacteria | Campylobacter | concisus | 0,304 | 0,000 |
| PWY.6386..UDP.N.acetylmuramoyl.pentapeptide.biosynthesis  .II..lysine.containing | Campylobacter | concisus | 0,286 | 0,000 |
| THRESYN.PWY..superpathway.of.L.threonine.biosynthesis | Actinomyces | graevenitzii | 0,611 | 0,000 |
| PWY.6549..L lutamine.biosynthesis.III.unclassified |  |  | 0,366 | 0,000 |
| PWY.6124..inosine.5..phosphate.biosynthesis.II | Atopobium | parvulum | 0,486 | 0,000 |
| NONMEVIPP.PWY..methylerythritol.phosphate.pathway.I | Prevotella | pallens | 0,463 | 0,000 |
| PWY.7221 uanosine.ribonucleotides.de.novo.biosynthesis | Prevotella | pallens | 0,560 | 0,000 |
| PWY.7851..coenzyme.A.biosynthesis.II..eukaryotic | Veillonella | atypica | 0,762 | 0,000 |
| UDPNAGSYN.PWY..UDP.N.acetyl.D lucosamine.biosynthesis.I | Leptotrichia | wadei | 0,652 | 0,000 |
| TCA..TCA.cycle.I..prokaryotic. |  |  | 0,543 | 0,000 |
| PWY.5097..L.lysine.biosynthesis.VI | Campylobacter | concisus | 0,361 | 0,000 |
| PWY.6936..seleno.amino.acid.biosynthesis..plants | Actinomyces | graevenitzii | 0,519 | 0,000 |
| PWY.6703..preQ0.biosynthesis.unclassified |  |  | 0,341 | 0,000 |
| PWY.2942..L.lysine.biosynthesis.III | Campylobacter | concisus | 0,464 | 0,000 |
| PWY0.862...5Z..dodecenoate.biosynthesis.I.unclassified |  |  | 0,476 | 0,000 |
| PWY.5686..UMP.biosynthesis.I | Selenomonas | flueggei | 0,246 | 0,000 |
| PWY.7790..UMP.biosynthesis.II | Selenomonas | flueggei | 0,246 | 0,000 |
| PWY.7791..UMP.biosynthesis.III | Selenomonas | flueggei | 0,246 | 0,000 |
| PWY.6163..chorismate.biosynthesis.from.3.dehydroquinate | Clostridiales_Family_XIII |  | 0,332 | 0,000 |
| PWY.6703..preQ0.biosynthesis | Campylobacter | concisus | 0,522 | 0,000 |
| HSERMETANA.PWY..L.methionine.biosynthesis.III | Lachnospiraceae | bacterium oral taxon 96 | 0,461 | 0,000 |
| ARO.PWY..chorismate.biosynthesis.I | Prevotella | pallens | 0,502 | 0,000 |
| PWY.7851..coenzyme.A.biosynthesis.II..eukaryotic | Prevotella | pallens | 0,658 | 0,000 |
| PWY.6386..UDP.N.acetylmuramoyl.pentapeptide.  biosynthesis.II..lysine.containing | Prevotella | sp oral taxon 306 | 0,665 | 0,000 |
| PWY.6385..peptidoglycan.biosynthesis.III..mycobacteria | Prevotella | pallens | 0,710 | 0,000 |
| VALSYN.PWY..L.valine.biosynthesis | Lachnospiraceae | bacterium oral taxon 96 | 0,414 | 0,000 |
| PWY.5097..L.lysine.biosynthesis.VI | Selenomonas | flueggei | 0,298 | 0,000 |
| DAPLYSINESYN.PWY..L.lysine.biosynthesis.I | Campylobacter | concisus | 0,338 | 0,000 |
| PWY.6121..5.aminoimidazole.ribonucleotide.biosynthesis.I | Selenomonas | flueggei | 0,318 | 0,000 |
| PWY.5686..UMP.biosynthesis.I | Prevotella | pallens | 0,479 | 0,000 |
| PWY.7790..UMP.biosynthesis.II | Prevotella | pallens | 0,479 | 0,000 |
| PWY.7791..UMP.biosynthesis.III | Prevotella | pallens | 0,479 | 0,000 |
| PWY66.409..superpathway.of.purine.nucleotide.salvage.unclassified |  |  | 0,320 | 0,000 |
| FASYN.ELONG.PWY..fatty.acid.elongation....saturated | Campylobacter | concisus | 0,520 | 0,000 |
| PWY.7664..oleate.biosynthesis.IV..anaerobic | Campylobacter | concisus | 0,529 | 0,000 |
| PWY.6282..palmitoleate.biosynthesis.I..from..5Z..dodec.5.enoate | Campylobacter | concisus | 0,536 | 0,000 |
| PWY.3841..folate.transformations.II..plants | Leptotrichia | wadei | 0,467 | 0,000 |
| PWY0.862...5Z..dodecenoate.biosynthesis.I | Campylobacter | concisus | 0,540 | 0,000 |
| P41.PWY..pyruvate.fermentation.to.acetate.and..S..lactate.I | Actinomyces | graevenitzii | 0,556 | 0,000 |
| PWY.5100..pyruvate.fermentation.to.acetate.and.lactate.II | Actinomyces | graevenitzii | 0,556 | 0,000 |
| PWY.6121..5.aminoimidazole.ribonucleotide.biosynthesis.I | Prevotella | pallens | 0,583 | 0,000 |
| PWY.1042.glycolysis.IV | Leptotrichia | wadei | 0,479 | 0,000 |
| PWY.6124..inosine.5..phosphate.biosynthesis.II | Prevotella | pallens | 0,681 | 0,000 |
| PWY.6147..6.hydroxymethyl.dihydropterin.diphosphate.biosynthesis.I | Selenomonas | flueggei | 0,264 | 0,000 |
| PWY.6630..superpathway.of.L.tyrosine.biosynthesis.unclassified |  |  | 0,379 | 0,000 |
| PWY.6823..molybdopterin.biosynthesis | Veillonella | atypica | 0,747 | 0,000 |
| PWY.5484.glycolysis.II..from.fructose.6.phosphate..unclassified |  |  | 0,330 | 0,000 |
| UNINTEGRATED | Prevotella | pallens | 0,528 | 0,000 |
| PEPTIDOGLYCANSYN.PWY..peptidoglycan.biosynthesis.  I..meso.diaminopimelate.containing | Prevotella | pallens | 0,733 | 0,000 |
| PWY.1042.glycolysis.IV | Prevotella | pallens | 0,630 | 0,000 |
| PWY.6317..D alactose.degradation.I..Leloir.pathway | Selenomonas | flueggei | 0,383 | 0,000 |
| PWY.5188..tetrapyrrole.biosynthesis.I..from lutamate..unclassified |  |  | 0,331 | 0,000 |
| ARO.PWY..chorismate.biosynthesis.I | Clostridiales_Family_XIII |  | 0,325 | 0,000 |
| PWY.6895..superpathway.of.thiamine.diphosphate.biosynthesis.II | Campylobacter | concisus | 0,492 | 0,000 |
| PWY.6386..UDP.N.acetylmuramoyl.pentapeptide.biosynthesis.II..lysine.containing | Prevotella | pallens | 0,740 | 0,000 |
| P461.PWY..hexitol.fermentation.to.lactate..formate..ethanol.and.acetate.unclassified |  |  | 0,592 | 0,000 |
| PWY.7199..pyrimidine.deoxyribonucleosides.salvage | Clostridiales_Family_XIII |  | 0,265 | 0,000 |
| PWY.7197..pyrimidine.deoxyribonucleotide.phosphorylation | Selenomonas | flueggei | 0,255 | 0,000 |
| HISTSYN.PWY..L.histidine.biosynthesis | Veillonella | atypica | 0,618 | 0,000 |
| PWY.7790..UMP.biosynthesis.II.unclassified |  |  | 0,192 | 0,000 |
| VALSYN.PWY..L.valine.biosynthesis | Actinomyces | graevenitzii | 0,591 | 0,000 |
| ANAEROFRUCAT.PWY..homolactic.fermentation.unclassified |  |  | 0,315 | 0,000 |
| COMPLETE.ARO.PWY..superpathway.of.aromatic.amino.acid.biosynthesis | Campylobacter | concisus | 0,246 | 0,000 |
| COLANSYN.PWY..colanic.acid.building.blocks.biosynthesis |  |  | 0,113 | 0,000 |
| PWY.6387..UDP.N.acetylmuramoyl.pentapeptide.biosynthesis  .I..meso.diaminopimelate.containing | Prevotella | pallens | 0,715 | 0,000 |
| BRANCHED.CHAIN.AA.SYN.PWY..superpathway  .of.branched.chain.amino.acid.biosynthesis | Campylobacter | concisus | 0,357 | 0,000 |
| PWY.7791..UMP.biosynthesis.III.unclassified |  |  | 0,197 | 0,000 |
| PWY.5384..sucrose.degradation.IV..sucrose.phosphorylase | Prevotella | pallens | 0,556 | 0,000 |
| NONMEVIPP.PWY..methylerythritol.phosphate.pathway.I | Atopobium | parvulum | 0,272 | 0,000 |
| PWY.6630..superpathway.of.L.tyrosine.biosynthesis | Campylobacter | concisus | 0,249 | 0,000 |
| G.glycolysis.glycolysis.I..from lucose.6.phosphate..unclassified |  |  | 0,324 | 0,000 |
| PWY.7199..pyrimidine.deoxyribonucleosides.salvage | Prevotella | pallens | 0,562 | 0,000 |
| UNINTEGRATED | Clostridiales_Family_XIII |  | 0,440 | 0,000 |
| PWY.724..superpathway.of.L.lysine..L.threonine.and.L.  methionine.biosynthesis.II.unclassified |  |  | 0,257 | 0,001 |
| PWY.5981..CDP.diacylglycerol.biosynthesis.III | Campylobacter | concisus | 0,266 | 0,001 |
| PWY.6628..superpathway.of.L.phenylalanine.biosynthesis | Campylobacter | concisus | 0,236 | 0,001 |
| VALSYN.PWY..L.valine.biosynthesis | Leptotrichia | wadei | 0,512 | 0,001 |
| COA.PWY..coenzyme.A.biosynthesis.I..prokaryotic | Prevotella | pallens | 0,513 | 0,001 |
| PWY.6163..chorismate.biosynthesis.from.3.dehydroquinate | Prevotella | pallens | 0,517 | 0,001 |
| PWY.6151..S.adenosyl.L.methionine.salvage.I | Solobacterium | moorei | 0,366 | 0,001 |
| HSERMETANA.PWY..L.methionine.biosynthesis.III | Atopobium | parvulum | 0,549 | 0,001 |
| COMPLETE.ARO.PWY..superpathway.of.aromatic.  amino.acid.biosynthesis.unclassified |  |  | 0,302 | 0,001 |
| NONOXIPENT.PWY..pentose.phosphate.pathway..  non.oxidative.branch..I | Selenomonas | flueggei | 0,348 | 0,001 |
| PWY.5973..cis.vaccenate.biosynthesis.unclassified |  |  | 0,262 | 0,001 |
| GLUCONEO.PWY luconeogenesis.I.unclassified |  |  | 0,359 | 0,001 |
| PWY.7229..superpathway.of.adenosine.nucleotides  .de.novo.biosynthesis.I | Veillonella | atypica | 0,585 | 0,001 |
| PWY.7953..UDP.N.acetylmuramoyl.pentapeptide.biosynthesis  .III..meso.diaminopimelate.containing | Prevotella | sp oral taxon 306 | 0,661 | 0,001 |
| PWY0.1296..purine.ribonucleosides.degradation | Atopobium | parvulum | 0,507 | 0,001 |
| PWY.6123..inosine.5..phosphate.biosynthesis.I | Campylobacter | concisus | 0,440 | 0,001 |
| NONOXIPENT.PWY..pentose.phosphate.pathway..non.oxidative.branch..I | Prevotella | pallens | 0,631 | 0,001 |
| GLUTORN.PWY..L.ornithine.biosynthesis.I | Selenomonas | flueggei | 0,287 | 0,001 |
| PWY0.1586..peptidoglycan.maturation..meso.diaminopimelate.containing | Prevotella | sp oral taxon 306 | 0,620 | 0,001 |
| PWY.5913..partial.TCA.cycle..obligate.autotrophs..unclassified |  |  | 0,379 | 0,001 |
| PWY.6147..6.hydroxymethyl.dihydropterin.diphosphate.biosynthesis.I | Campylobacter | concisus | 0,443 | 0,001 |
| PWY.6527..stachyose.degradation | Selenomonas | flueggei | 0,415 | 0,001 |
| HEMESYN2.PWY..heme.b.biosynthesis.II..oxygen.independent..unclassified |  |  | 0,413 | 0,001 |
| PWY.5188..tetrapyrrole.biosynthesis.I..from lutamate | Actinomyces | graevenitzii | 0,528 | 0,001 |
| COBALSYN.PWY..superpathway.of.adenosylcobalamin.salvage.from.cobinamide.I | Clostridiales_Family_XIII |  | 0,337 | 0,001 |
| PWY.6122..5.aminoimidazole.ribonucleotide.biosynthesis.II | Selenomonas | flueggei | 0,285 | 0,001 |
| PWY.6277..superpathway.of.5.aminoimidazole.ribonucleotide.biosynthesis | Selenomonas | flueggei | 0,285 | 0,001 |
| PWY.6700..queuosine.biosynthesis.I..de.novo | Selenomonas | flueggei | 0,303 | 0,001 |
| PWY.6122..5.aminoimidazole.ribonucleotide.biosynthesis.II | Lachnospiraceae | bacterium oral taxon 96 | 0,479 | 0,001 |
| PWY.6277..superpathway.of.5.aminoimidazole.ribonucleotide.biosynthesis | Lachnospiraceae | bacterium oral taxon 96 | 0,479 | 0,001 |
| PWY.5188..tetrapyrrole.biosynthesis.I..from lutamate | Leptotrichia | wadei | 0,477 | 0,001 |
| ARGSYN.PWY..L.arginine.biosynthesis.I..via.L.ornithine | Selenomonas | flueggei | 0,318 | 0,001 |
| ARGSYNBSUB.PWY..L.arginine.biosynthesis.II..acetyl.cycle | Selenomonas | flueggei | 0,318 | 0,001 |
| PWY.5840..superpathway.of.menaquinol.7.biosynthesis.unclassified |  |  | 0,453 | 0,001 |
| PWY.7357..thiamine.phosphate.formation.from.pyrithiamine.and.oxythiamine..yeast | Selenomonas | flueggei | 0,327 | 0,001 |
| PWY.6609..adenine.and.adenosine.salvage.III | Atopobium | parvulum | 0,568 | 0,001 |
| PWY.7953..UDP.N.acetylmuramoyl.pentapeptide.  biosynthesis.III..meso.diaminopimelate.containing | Prevotella | pallens | 0,734 | 0,001 |
| CALVIN.PWY..Calvin.Benson.Bassham.cycle.unclassified |  |  | 0,254 | 0,001 |
| UNINTEGRATED.unclassified |  |  | 0,078 | 0,001 |
| PWY.5686..UMP.biosynthesis.I.unclassified |  |  | 0,188 | 0,001 |
| PWY.6126..superpathway.of.adenosine.nucleotides  .de.novo.biosynthesis.II | Veillonella | atypica | 0,566 | 0,001 |
| PWY.8178..pentose.phosphate.pathway..non.oxidative.branch..II | Selenomonas | flueggei | 0,357 | 0,001 |
| COA.PWY.1..superpathway.of.coenzyme.A.biosynthesis.III..mammals | Atopobium | parvulum | 0,380 | 0,001 |
| VALSYN.PWY..L.valine.biosynthesis | Selenomonas | flueggei | 0,338 | 0,001 |
| PWY.7228..superpathway.of uanosine.nucleotides.de.novo.biosynthesis.I | Actinomyces | graevenitzii | 0,537 | 0,001 |
| COA.PWY.1..superpathway.of.coenzyme.A.biosynthesis.III..mammals | Leptotrichia | wadei | 0,249 | 0,001 |
| PWY.5121..superpathway.of eranylgeranyl.diphosphate  .biosynthesis.II..via.MEP | Campylobacter | concisus | 0,389 | 0,001 |
| DTDPRHAMSYN.PWY..dTDP..beta..L.rhamnose.biosynthesis | Atopobium | parvulum | 0,505 | 0,001 |
| PWY.6700..queuosine.biosynthesis.I..de.novo..unclassified |  |  | 0,246 | 0,001 |
| OANTIGEN.PWY..O.antigen.building.blocks.biosynthesis..E..coli. |  |  | 0,048 | 0,001 |
| COA.PWY..coenzyme.A.biosynthesis.I..prokaryotic | Veillonella | atypica | 0,675 | 0,001 |
| PWY.7254..TCA.cycle.VII..acetate.producers. |  |  | 0,372 | 0,001 |
| PWY.7238..sucrose.biosynthesis.II | Prevotella | pallens | 0,539 | 0,001 |
| PWY.2941..L.lysine.biosynthesis.II.unclassified |  |  | 0,364 | 0,001 |
| CALVIN.PWY..Calvin.Benson.Bassham.cycle | Campylobacter | concisus | 0,462 | 0,001 |
| PWY.2942..L.lysine.biosynthesis.III | Veillonella | atypica | 0,585 | 0,001 |
| RIBOSYN2.PWY..flavin.biosynthesis.I..bacteria.and.plants. |  |  | 0,064 | 0,001 |
| PWY.6387..UDP.N.acetylmuramoyl.pentapeptide.  biosynthesis.I..meso.diaminopimelate.containing | Actinomyces | graevenitzii | 0,533 | 0,001 |
| PWY.7851..coenzyme.A.biosynthesis.II..eukaryotic. |  |  | 0,048 | 0,001 |
| PWY.6386..UDP.N.acetylmuramoyl.pentapeptide.  biosynthesis.II..lysine.containing | Actinomyces | graevenitzii | 0,532 | 0,001 |
| COA.PWY..coenzyme.A.biosynthesis.I..prokaryotic. |  |  | 0,047 | 0,001 |
| COA.PWY..coenzyme.A.biosynthesis.I..prokaryotic | Prevotella | sp oral taxon 306 | 0,761 | 0,001 |
| X1CMET2.PWY..folate.transformations.III..E..coli | Prevotella | jejuni | 0,680 | 0,001 |
| ARO.PWY..chorismate.biosynthesis.I | Lachnospiraceae | bacterium oral taxon 96 | 0,325 | 0,001 |
| PWY.5941 lycogen.degradation.II | Prevotella | pallens | 0,592 | 0,001 |
| RIBOSYN2.PWY..flavin.biosynthesis.I..bacteria.and.plants | Prevotella | sp oral taxon 306 | 0,463 | 0,001 |
| PWY.5097..L.lysine.biosynthesis.VI | Veillonella | atypica | 0,595 | 0,001 |
| PWY.5973..cis.vaccenate.biosynthesis | Veillonella | atypica | 0,614 | 0,001 |
| PWY.6700..queuosine.biosynthesis.I..de.novo | Clostridiales_Family_XIII |  | 0,283 | 0,001 |
| TRNA.CHARGING.PWY..tRNA.charging | Clostridiales_Family_XIII |  | 0,243 | 0,001 |
| PWY.5971..palmitate.biosynthesis..type.II.fatty.acid.synthase.. |  |  | 0,512 | 0,001 |
| PWY.5973..cis.vaccenate.biosynthesis | Prevotella | jejuni | 0,661 | 0,001 |
| PEPTIDOGLYCANSYN.PWY..peptidoglycan.biosynthesis.  I..meso.diaminopimelate.containing | Clostridiales_Family_XIII |  | 0,274 | 0,001 |
| PWY.6124..inosine.5..phosphate.biosynthesis.II | Selenomonas | flueggei | 0,315 | 0,001 |
| PANTO.PWY..phosphopantothenate.biosynthesis.I | Actinomyces | graevenitzii | 0,483 | 0,001 |
| PWY.6122..5.aminoimidazole.ribonucleotide.biosynthesis.II | Clostridiales_Family_XIII |  | 0,343 | 0,001 |
| PWY.6277..superpathway.of.5.aminoimidazole.ribonucleotide.biosynthesis | Clostridiales_Family_XIII |  | 0,343 | 0,001 |
| PWY.6385..peptidoglycan.biosynthesis.III..mycobacteria | Prevotella | sp oral taxon 306 | 0,607 | 0,001 |
| PWY.7851..coenzyme.A.biosynthesis.II..eukaryotic | Leptotrichia | wadei | 0,246 | 0,001 |
|  |  |  |  |  |
| PWY66.429..fatty.acid.biosynthesis.initiation..mitochondria | Clostridiales_Family_XIII |  | 0,297 | 0,001 |
| PWY.7197..pyrimidine.deoxyribonucleotide.phosphorylation | Actinomyces | graevenitzii | 0,519 | 0,001 |
| PWY.6151..S.adenosyl.L.methionine.salvage.I | Prevotella | sp oral taxon 306 | 0,548 | 0,001 |
| P461.PWY..hexitol.fermentation.to.lactate..formate..ethanol.and.acetate | Selenomonas | flueggei | 0,371 | 0,001 |
| PWY.5005..biotin.biosynthesis.II.unclassified |  |  | 0,430 | 0,001 |
| PEPTIDOGLYCANSYN.PWY..peptidoglycan.biosynthesis.  I..meso.diaminopimelate.containing | Actinomyces | graevenitzii | 0,423 | 0,001 |
| PWY.3841..folate.transformations.II..plants | Clostridiales_Family_XIII |  | 0,401 | 0,001 |
| ARO.PWY..chorismate.biosynthesis.I | Campylobacter | concisus | 0,370 | 0,001 |
| DTDPRHAMSYN.PWY..dTDP..beta..L.rhamnose.biosynthesis | Prevotella | pallens | 0,565 | 0,001 |
| PWY.1042.glycolysis.IV | Veillonella | atypica | 0,630 | 0,001 |
| PWY.7953..UDP.N.acetylmuramoyl.pentapeptide.biosynthesis.  III..meso.diaminopimelate.containing | Veillonella | atypica | 0,598 | 0,001 |
| PWY.7663 ondoate.biosynthesis..anaerobic | Prevotella | jejuni | 0,663 | 0,001 |
| PWY.6125..superpathway.of uanosine.nucleotides.  de.novo.biosynthesis.II | Actinomyces | graevenitzii | 0,520 | 0,001 |
| PWY.5686..UMP.biosynthesis.I | Veillonella | atypica | 0,636 | 0,001 |
| PWY.7790..UMP.biosynthesis.II | Veillonella | atypica | 0,636 | 0,001 |
| PWY.7791..UMP.biosynthesis.III | Veillonella | atypica | 0,636 | 0,001 |
| PWY.6609..adenine.and.adenosine.salvage.III | Clostridiales_Family_XIII |  | 0,330 | 0,001 |
| PWY.7663 ondoate.biosynthesis..anaerobic | Veillonella | atypica | 0,622 | 0,001 |
| NONMEVIPP.PWY..methylerythritol.phosphate.pathway.I | Actinomyces | graevenitzii | 0,436 | 0,001 |
| PWY.7197..pyrimidine.deoxyribonucleotide.phosphorylation | Actinomyces | sp S6 Spd3 | 0,439 | 0,001 |
| HSERMETANA.PWY..L.methionine.biosynthesis.III | Actinomyces | sp S6 Spd3 | 0,396 | 0,001 |
| PWY.8004..Entner.Doudoroff.pathway.I.unclassified |  |  | 0,348 | 0,001 |
| SER LYSYN.PWY..superpathway.of.L.serine.and lycine.biosynthesis.I | Clostridiales_Family_XIII |  | 0,414 | 0,001 |
| PWY.6703..preQ0.biosynthesis | Lachnospiraceae | bacterium oral taxon 96 | 0,386 | 0,001 |
| PWY.7221 uanosine.ribonucleotides.de.novo.biosynthesis | Actinomyces | graevenitzii | 0,503 | 0,001 |
| PWY.7228..superpathway.of uanosine.nucleotides.de.novo.biosynthesis.I | Veillonella | atypica | 0,587 | 0,001 |
| PWY.7208..superpathway.of.pyrimidine.nucleobases.salvage | Veillonella | atypica | 0,571 | 0,001 |
| NAGLIPASYN.PWY..lipid.IVA.biosynthesis..E..coli | Campylobacter | concisus | 0,412 | 0,002 |
| PWY.8073..lipid.IVA.biosynthesis..P..putida | Campylobacter | concisus | 0,412 | 0,002 |
| PWY.2942..L.lysine.biosynthesis.III | Lachnospiraceae | bacterium oral taxon 96 | 0,314 | 0,002 |
| PWY.5686..UMP.biosynthesis.I | Lachnospiraceae | bacterium oral taxon 96 | 0,291 | 0,002 |
| PWY.7790..UMP.biosynthesis.II | Lachnospiraceae | bacterium oral taxon 96 | 0,291 | 0,002 |
| PWY.7791..UMP.biosynthesis.III | Lachnospiraceae | bacterium oral taxon 96 | 0,291 | 0,002 |
| PWY.5188..tetrapyrrole.biosynthesis.I..from lutamate | Campylobacter | concisus | 0,392 | 0,002 |
| PWY.6700..queuosine.biosynthesis.I..de.novo | Prevotella | pallens | 0,620 | 0,002 |
| PEPTIDOGLYCANSYN.PWY..peptidoglycan.biosynthesis  .I..meso.diaminopimelate.containing. |  |  | 0,049 | 0,002 |
| PWY.6124..inosine.5..phosphate.biosynthesis.II | Campylobacter | concisus | 0,421 | 0,002 |
| CALVIN.PWY..Calvin.Benson.Bassham.cycle | Prevotella | pallens | 0,641 | 0,002 |
| PWY.5686..UMP.biosynthesis.I | Clostridiales_Family_XIII |  | 0,259 | 0,002 |
| PWY.7790..UMP.biosynthesis.II | Clostridiales_Family_XIII |  | 0,259 | 0,002 |
| PWY.7791..UMP.biosynthesis.III | Clostridiales_Family_XIII |  | 0,259 | 0,002 |
| PWY.6823..molybdopterin.biosynthesis | Selenomonas | flueggei | 0,357 | 0,002 |
| PWY.6163..chorismate.biosynthesis.from.3.dehydroquinate.unclassified |  |  | 0,173 | 0,002 |
| PWY.6700..queuosine.biosynthesis.I..de.novo | Lachnospiraceae | bacterium oral taxon 96 | 0,365 | 0,002 |
| HEME.BIOSYNTHESIS.II.1..heme.b.biosynthesis.  V..aerobic..unclassified |  |  | 0,511 | 0,002 |
| PWY.724..superpathway.of.L.lysine..L.threonine.and.L.  methionine.biosynthesis.II | Veillonella | atypica | 0,552 | 0,002 |
| NONMEVIPP.PWY..methylerythritol.phosphate.pathway.I | Campylobacter | concisus | 0,354 | 0,002 |
| PWY.7221 uanosine.ribonucleotides.de.novo.biosynthesis | Selenomonas | flueggei | 0,324 | 0,002 |
| PWY.5686..UMP.biosynthesis.I | Prevotella | jejuni | 0,602 | 0,002 |
| PWY.7790..UMP.biosynthesis.II | Prevotella | jejuni | 0,602 | 0,002 |
| PWY.7791..UMP.biosynthesis.III | Prevotella | jejuni | 0,602 | 0,002 |
| X1CMET2.PWY..folate.transformations.III..E..coli | Prevotella | sp oral taxon 306 | 0,496 | 0,002 |
| PWY.6387..UDP.N.acetylmuramoyl.pentapeptide.  biosynthesis.I..meso.diaminopimelate.containing. |  |  | 0,049 | 0,002 |
| PWY.6147..6.hydroxymethyl.dihydropterin.  diphosphate.biosynthesis.I | Prevotella | sp oral taxon 306 | 0,590 | 0,002 |
| PWY0.1298..superpathway.of.pyrimidine.  deoxyribonucleosides.degradation.unclassified |  |  | 0,258 | 0,002 |
| PWY.5097..L.lysine.biosynthesis.VI |  |  | 0,052 | 0,002 |
| COA.PWY..coenzyme.A.biosynthesis.I..prokaryotic | Clostridiales_Family_XIII |  | 0,384 | 0,002 |
| PWY.6125..superpathway.of uanosine.nucleotides.de.novo.biosynthesis.II | Veillonella | atypica | 0,576 | 0,002 |
| HISTSYN.PWY..L.histidine.biosynthesis.unclassified |  |  | 0,361 | 0,002 |
| DAPLYSINESYN.PWY..L.lysine.biosynthesis.I.unclassified |  |  | 0,296 | 0,002 |
| PWY0.1241..ADP.L lycero..beta..D.manno.heptose.biosynthesis | Veillonella | atypica | 0,675 | 0,002 |
| PWY.7197..pyrimidine.deoxyribonucleotide.phosphorylation | Veillonella | atypica | 0,477 | 0,002 |
| HEME.BIOSYNTHESIS.II..heme.b.biosynthesis.I..aerobic.. |  |  | 0,350 | 0,002 |
| PYRIDNUCSYN.PWY..NAD.de.novo.biosynthesis.I..from.aspartate. |  |  | 0,093 | 0,002 |
| PWY0.1296..purine.ribonucleosides.degradation | Veillonella | atypica | 0,609 | 0,002 |
| RIBOSYN2.PWY..flavin.biosynthesis.I..bacteria.and.plants.. |  |  | 0,314 | 0,002 |
| PWY.5130..2.oxobutanoate.degradation.I | Veillonella | atypica | 0,563 | 0,002 |
| PWY.6163..chorismate.biosynthesis.from.3.dehydroquinate | Lachnospiraceae | bacterium oral taxon 96 | 0,400 | 0,002 |
| PWY.3841..folate.transformations.II..plants | Prevotella | sp oral taxon 306 | 0,658 | 0,002 |
| GLYCOGENSYNTH.PWY lycogen.biosynthesis.I.  .from.ADP.D lucose | Lachnospiraceae | bacterium oral taxon 96 | 0,361 | 0,002 |
| PWY.7977..L.methionine.biosynthesis.IV | Prevotella | sp oral taxon 306 | 0,559 | 0,002 |
| PWY.3841..folate.transformations.II..plants | Actinomyces | graevenitzii | 0,450 | 0,002 |
| PWY.6147..6.hydroxymethyl.dihydropterin.diphosphate.biosynthesis.I | Prevotella | jejuni | 0,635 | 0,002 |
| SER LYSYN.PWY..superpathway.of.L.serine.and lycine.biosynthesis.I | Campylobacter | concisus | 0,404 | 0,002 |
| PWY.6897..thiamine.diphosphate.salvage.II | Clostridiales_Family_XIII |  | 0,286 | 0,002 |
| PWY.6609..adenine.and.adenosine.salvage.III | Lachnospiraceae | bacterium oral taxon 96 | 0,448 | 0,002 |
| PWY.6386..UDP.N.acetylmuramoyl.pentapeptide.biosynthesis  .II..lysine.containing | Veillonella | atypica | 0,530 | 0,002 |
| PWY.5097..L.lysine.biosynthesis.VI | Prevotella | sp oral taxon 306 | 0,583 | 0,003 |
| PWY.6151..S.adenosyl.L.methionine.salvage.I | Prevotella | jejuni | 0,666 | 0,003 |
| PWY.5695..inosine.5..phosphate.degradation | Clostridiales_Family_XIII |  | 0,366 | 0,003 |
| PWY.6163..chorismate.biosynthesis.from.3.dehydroquinate | Prevotella | jejuni | 0,607 | 0,003 |
| PWY0.1586..peptidoglycan.maturation..meso.diaminopimelate.containing | Selenomonas | sputigena | 0,335 | 0,003 |
| PWY.7220..adenosine.deoxyribonucleotides.de.novo.biosynthesis.II | Veillonella | atypica | 0,530 | 0,003 |
| PWY.7222 uanosine.deoxyribonucleotides.de.novo.biosynthesis.II | Veillonella | atypica | 0,530 | 0,003 |
| PWY.5103..L.isoleucine.biosynthesis.III | Campylobacter | concisus | 0,472 | 0,003 |
| ILEUSYN.PWY..L.isoleucine.biosynthesis.I..from.threonine | Campylobacter | concisus | 0,476 | 0,003 |
| PWY.6123..inosine.5..phosphate.biosynthesis.I | Actinomyces | graevenitzii | 0,476 | 0,003 |
| PWY.5897..superpathway.of.menaquinol.11.biosynthesis.unclassified |  |  | 0,401 | 0,003 |
| PWY.5898..superpathway.of.menaquinol.12.biosynthesis.unclassified |  |  | 0,401 | 0,003 |
| PWY.5899..superpathway.of.menaquinol.13.biosynthesis.unclassified |  |  | 0,401 | 0,003 |
| PWY.5188..tetrapyrrole.biosynthesis.I..from lutamate | Lachnospiraceae | bacterium oral taxon 96 | 0,300 | 0,003 |
| NONMEVIPP.PWY..methylerythritol.phosphate.pathway.I | Prevotella | jejuni | 0,640 | 0,003 |
| PWY.5189..tetrapyrrole.biosynthesis.II..from lycine | Campylobacter | concisus | 0,385 | 0,003 |
| PWY.7357..thiamine.phosphate.formation.from.pyrithiamine.and.oxythiamine.. | Clostridiales_Family_XIII |  | 0,360 | 0,003 |
| PWY.6317..D alactose.degradation.I..Leloir.pathway | Atopobium | parvulum | 0,174 | 0,003 |
| PWY.6123..inosine.5..phosphate.biosynthesis.I | Atopobium | parvulum | 0,427 | 0,003 |
| PWY.5838..superpathway.of.menaquinol.8.biosynthesis.I.unclassified |  |  | 0,383 | 0,003 |
| PWY.7111..pyruvate.fermentation.to.isobutanol..engineered..unclassified |  |  | 0,361 | 0,003 |
| PWY66.429..fatty.acid.biosynthesis.initiation..mitochondria | Veillonella | atypica | 0,535 | 0,003 |
| COA.PWY.1..superpathway.of.coenzyme.A.biosynthesis.III..mammals. |  |  | 0,044 | 0,003 |
| PWY.5505..L lutamate.and.L lutamine.biosynthesis |  |  | 0,198 | 0,003 |
| UNINTEGRATED | Selenomonas | flueggei | 0,403 | 0,003 |
| PEPTIDOGLYCANSYN.PWY..peptidoglycan.biosynthesis.I..  meso.diaminopimelate.containing..unclassified |  |  | 0,159 | 0,003 |
| PWY.6823..molybdopterin.biosynthesis | Campylobacter | concisus | 0,427 | 0,003 |
| PWY.6151..S.adenosyl.L.methionine.salvage.I | Actinomyces | graevenitzii | 0,429 | 0,003 |
| PWY.7221 uanosine.ribonucleotides.de.novo.biosynthesis | Prevotella | jejuni | 0,727 | 0,003 |
| NONOXIPENT.PWY..pentose.phosphate.pathway..non.oxidative.branch..I | Lachnospiraceae | bacterium oral taxon 96 | 0,329 | 0,003 |
| ANAG.glycolysis.PWY.glycolysis.III..from lucose | Prevotella | jejuni | 0,727 | 0,003 |
| PWY.7208..superpathway.of.pyrimidine.nucleobases.salvage | Actinomyces | graevenitzii | 0,477 | 0,003 |
| PWY.1042.glycolysis.IV.unclassified |  |  | 0,260 | 0,003 |
| PWY.5686..UMP.biosynthesis.I | Actinomyces | graevenitzii | 0,429 | 0,003 |
| PWY.7220..adenosine.deoxyribonucleotides.de.novo.biosynthesis.II | Actinomyces | graevenitzii | 0,492 | 0,003 |
| PWY.7222 uanosine.deoxyribonucleotides.de.novo.biosynthesis.II | Actinomyces | graevenitzii | 0,492 | 0,003 |
| ASPASN.PWY..superpathway.of.L.aspartate.and.L  .asparagine.biosynthesis.unclassified |  |  | 0,259 | 0,003 |
| COA.PWY..coenzyme.A.biosynthesis.I..prokaryotic | Atopobium | parvulum | 0,418 | 0,003 |
| HEMESYN2.PWY..heme.b.biosynthesis.II..oxygen.independent | Campylobacter | concisus | 0,495 | 0,003 |
| PWY.6385..peptidoglycan.biosynthesis.III..mycobacteria | Veillonella | atypica | 0,522 | 0,003 |
| PWY.7851..coenzyme.A.biosynthesis.II..eukaryotic | Lachnospiraceae | bacterium oral taxon 96 | 0,310 | 0,003 |
| HSERMETANA.PWY..L.methionine.biosynthesis.III | Campylobacter | concisus | 0,373 | 0,004 |
| PWY.6386..UDP.N.acetylmuramoyl.pentapeptide.biosynthesis  .II..lysine.containing. |  |  | 0,048 | 0,004 |
| UNINTEGRATED | Atopobium | parvulum | 0,496 | 0,004 |
| PWY.7851..coenzyme.A.biosynthesis.II..eukaryotic | Prevotella | jejuni | 0,609 | 0,004 |
| PEPTIDOGLYCANSYN.PWY..peptidoglycan.biosynthesis.I..  meso.diaminopimelate.containing | Prevotella | sp oral taxon 306 | 0,584 | 0,004 |
| HSERMETANA.PWY..L.methionine.biosynthesis.III | Selenomonas | sputigena | 0,264 | 0,004 |
| GLUCONEO.PWY luconeogenesis.I |  |  | 0,049 | 0,004 |
| PWY.6897..thiamine.diphosphate.salvage.II | Campylobacter | concisus | 0,430 | 0,004 |
| PWY.7663 ondoate.biosynthesis..anaerobic | Campylobacter | concisus | 0,389 | 0,004 |
| PWY66.429..fatty.acid.biosynthesis.initiation..mitochondria | Campylobacter | concisus | 0,436 | 0,004 |
| UNMAPPED |  |  | -0,068 | 0,004 |
| PWY.6386..UDP.N.acetylmuramoyl.pentapeptide.  biosynthesis.II..lysine.containing | Lachnospiraceae | bacterium oral taxon 96 | 0,316 | 0,004 |
| PWY.8178..pentose.phosphate.pathway..non.oxidative.branch..II | Prevotella | pallens | 0,494 | 0,004 |
| PWY.6121..5.aminoimidazole.ribonucleotide.biosynthesis.I | Actinomyces | graevenitzii | 0,456 | 0,004 |
| PWY.6387..UDP.N.acetylmuramoyl.pentapeptide.biosynthesis  .I..meso.diaminopimelate.containing | Lachnospiraceae | bacterium oral taxon 96 | 0,317 | 0,004 |
| PWY.6936..seleno.amino.acid.biosynthesis..plants | Lachnospiraceae | bacterium oral taxon 96 | 0,324 | 0,004 |
| PEPTIDOGLYCANSYN.PWY..peptidoglycan.biosynthesis  .I..meso.diaminopimelate.containing | Veillonella | atypica | 0,513 | 0,004 |
| COBALSYN.PWY..superpathway.of.adenosylcobalamin.  salvage.from.cobinamide.I |  |  | 0,216 | 0,004 |
| ARGSYN.PWY..L.arginine.biosynthesis.I..via.L.ornithine | Lachnospiraceae | bacterium oral taxon 96 | 0,316 | 0,004 |
| COA.PWY..coenzyme.A.biosynthesis.I..prokaryotic | Lachnospiraceae | bacterium oral taxon 96 | 0,405 | 0,004 |
| PWY.1042.glycolysis.IV | Prevotella | sp oral taxon 306 | 0,552 | 0,004 |
| PWY.2942..L.lysine.biosynthesis.III.unclassified |  |  | 0,194 | 0,004 |
| PWY.5989..stearate.biosynthesis.II..bacteria.and.plants..unclassified |  |  | 0,217 | 0,004 |
| PWY.5030..L.histidine.degradation.III | Prevotella | jejuni | 0,647 | 0,004 |
| RHAMCAT.PWY..L.rhamnose.degradation.I.unclassified |  |  | 0,514 | 0,004 |
| UDPNAGSYN.PWY..UDP.N.acetyl.D lucosamine.biosynthesis.I | Campylobacter | concisus | 0,399 | 0,004 |
| PWY.5686..UMP.biosynthesis.I | Prevotella | sp oral taxon 306 | 0,567 | 0,004 |
| PWY.7790..UMP.biosynthesis.II | Prevotella | sp oral taxon 306 | 0,567 | 0,004 |
| PWY.7791..UMP.biosynthesis.III | Prevotella | sp oral taxon 306 | 0,567 | 0,004 |
| PWY.5097..L.lysine.biosynthesis.VI | Prevotella | melaninogenica | 0,561 | 0,004 |
| PWY0.1241..ADP.L lycero..beta..D.manno.heptose.biosynthesis | Campylobacter | concisus | 0,384 | 0,004 |
| NONMEVIPP.PWY..methylerythritol.phosphate.pathway.I | Veillonella | atypica | 0,547 | 0,004 |
| PWY.6387..UDP.N.acetylmuramoyl.pentapeptide.biosynthesis  .I..meso.diaminopimelate.containing | Prevotella | sp | 0,583 | 0,005 |
| HISDEG.PWY..L.histidine.degradation.I | Prevotella | jejuni | 0,664 | 0,005 |
| PWY.6386..UDP.N.acetylmuramoyl.pentapeptide.  biosynthesis.II..lysine.containing | Prevotella | jejuni | 0,664 | 0,005 |
| PWY.6317..D alactose.degradation.I..Leloir.pathway | Oribacterium | parvum | 0,213 | 0,005 |
| PWY.6385..peptidoglycan.biosynthesis.III..mycobacteria | Atopobium | parvulum | 0,272 | 0,005 |
| PWY.7221 uanosine.ribonucleotides.de.novo.biosynthesis | Selenomonas | sputigena | 0,213 | 0,005 |
| PEPTIDOGLYCANSYN.PWY..peptidoglycan.biosynthesis  .I..meso.diaminopimelate.containing | Atopobium | parvulum | 0,269 | 0,005 |
| PWY.1042.glycolysis.IV | Prevotella | jejuni | 0,721 | 0,005 |
| COA.PWY.1..superpathway.of.coenzyme.A.biosynthesis.III.. | Lachnospiraceae | bacterium oral taxon 96 | 0,290 | 0,005 |
| ARO.PWY..chorismate.biosynthesis.I |  |  | 0,042 | 0,005 |
| BRANCHED.CHAIN.AA.SYN.PWY..superpathway.of.  branched.chain.amino.acid.biosynthesis.unclassified |  |  | 0,293 | 0,005 |
| PWY.7221 uanosine.ribonucleotides.de.novo.biosynthesis | Prevotella | sp oral taxon 306 | 0,599 | 0,005 |
| ANAG.glycolysis.PWY.glycolysis.III..from lucose..unclassified |  |  | 0,219 | 0,005 |
| PWY.6609..adenine.and.adenosine.salvage.III | Veillonella | atypica | 0,589 | 0,005 |
| PWY.6936..seleno.amino.acid.biosynthesis..plants | Veillonella | atypica | 0,581 | 0,005 |
| PWY.7221 uanosine.ribonucleotides.de.novo.biosynthesis | Campylobacter | concisus | 0,349 | 0,005 |
| PWY.6700..queuosine.biosynthesis.I..de.novo | Veillonella | atypica | 0,551 | 0,005 |
| PWY.6387..UDP.N.acetylmuramoyl.pentapeptide.  biosynthesis.I..meso.diaminopimelate.containing..unclassified |  |  | 0,150 | 0,005 |
| PWY.6125..superpathway.of uanosine.nucleotides  .de.novo.biosynthesis.II | Campylobacter | concisus | 0,404 | 0,005 |
| PWY.5097..L.lysine.biosynthesis.VI | Prevotella | jejuni | 0,556 | 0,006 |
| PWY.7221 uanosine.ribonucleotides.de.novo.biosynthesis | Actinomyces | sp S6 Spd3 | 0,432 | 0,006 |
| PWY.7953..UDP.N.acetylmuramoyl.pentapeptide.  biosynthesis.III..meso.diaminopimelate.containing | Prevotella | jejuni | 0,699 | 0,006 |
| PWY.6123..inosine.5..phosphate.biosynthesis.I | Veillonella | atypica | 0,594 | 0,006 |
| PWY.6122..5.aminoimidazole.ribonucleotide.biosynthesis.II | Actinomyces | graevenitzii | 0,423 | 0,006 |
| PWY.6277..superpathway.of.5.aminoimidazole  .ribonucleotide.biosynthesis | Actinomyces | graevenitzii | 0,423 | 0,006 |
| PWY.1042.glycolysis.IV |  |  | 0,046 | 0,006 |
| UNINTEGRATED | Lachnospiraceae | bacterium oral taxon 96 | 0,551 | 0,006 |
| PWY.7371..1.4.dihydroxy.6.naphthoate.biosynthesis.II | Campylobacter | concisus | 0,372 | 0,006 |
| PWY.8131..5..deoxyadenosine.degradation.II |  |  | 0,391 | 0,006 |
| DTDPRHAMSYN.PWY..dTDP..beta..L.rhamnose.biosynthesis | Actinomyces | graevenitzii | 0,417 | 0,006 |
| PWY.6385..peptidoglycan.biosynthesis.III..mycobacteria..unclassified |  |  | 0,158 | 0,006 |
| PWY.5981..CDP.diacylglycerol.biosynthesis.III.unclassified |  |  | 0,330 | 0,006 |
| PYRIDNUCSYN.PWY..NAD.de.novo.biosynthesis.I..from.aspartate | Prevotella | melaninogenica | 0,396 | 0,006 |
| PWY.6121..5.aminoimidazole.ribonucleotide.biosynthesis.I.unclassified |  |  | 0,165 | 0,006 |
| PWY.6163..chorismate.biosynthesis.from.3.dehydroquinate | Prevotella | sp oral taxon 306 | 0,512 | 0,006 |
| ARO.PWY..chorismate.biosynthesis.I | Veillonella | atypica | 0,424 | 0,006 |
| PWY.5695..inosine.5..phosphate.degradation | Veillonella | atypica | 0,539 | 0,006 |
| PWY.6387..UDP.N.acetylmuramoyl.pentapeptide.  biosynthesis.I..meso.diaminopimelate.containing | Veillonella | atypica | 0,498 | 0,006 |
| ANAG.glycolysis.PWY.glycolysis.III..from lucose | Prevotella | sp oral taxon 306 | 0,528 | 0,007 |
| PWY.5920..superpathway.of.heme.b.biosynthesis.from lycine. |  |  | 0,273 | 0,007 |
| DTDPRHAMSYN.PWY..dTDP..beta..L.rhamnose.biosynthesis | Lachnospiraceae | bacterium oral taxon 96 | 0,405 | 0,007 |
| PHOSLIPSYN.PWY..superpathway.of.phospholipid.  biosynthesis.I..bacteria..unclassified |  |  | 0,342 | 0,007 |
| PWY.6385..peptidoglycan.biosynthesis.III..mycobacteria. |  |  | 0,044 | 0,007 |
| HISTSYN.PWY..L.histidine.biosynthesis | Campylobacter | concisus | 0,194 | 0,007 |
| PWY.6700..queuosine.biosynthesis.I..de.novo | Tannerella | sp oral taxon HOT 286 | 0,422 | 0,007 |
| PWY.6121..5.aminoimidazole.ribonucleotide.biosynthesis.I | Clostridiales_Family_XIII |  | 0,303 | 0,007 |
| UNINTEGRATED | Prevotella | jejuni | 0,547 | 0,007 |
| PWY.5667..CDP.diacylglycerol.biosynthesis.I.unclassified |  |  | 0,260 | 0,007 |
| DTDPRHAMSYN.PWY..dTDP..beta..L.rhamnose.biosynthesis | Prevotella | jejuni | 0,711 | 0,007 |
| PWY.6749..CMP.legionaminate.biosynthesis.I | Campylobacter | concisus | 0,375 | 0,007 |
| PWY.7208..superpathway.of.pyrimidine.nucleobases.salvage | Campylobacter | concisus | 0,407 | 0,007 |
| PWY.6700..queuosine.biosynthesis.I..de.novo | Prevotella | jejuni | 0,614 | 0,007 |
| P42.PWY..incomplete.reductive.TCA.cycle |  |  | 0,107 | 0,007 |
| PWY.3841..folate.transformations.II..plants | Veillonella | atypica | 0,454 | 0,007 |
| PWY.1269..CMP.3.deoxy.D.manno.octulosonate.biosynthesis | Campylobacter | concisus | 0,407 | 0,007 |
| PWY.6387..UDP.N.acetylmuramoyl.pentapeptide.  biosynthesis.I..meso.diaminopimelate.containing | Prevotella | jejuni | 0,688 | 0,007 |
| PWY.7357..thiamine.phosphate.formation.from.  pyrithiamine.and.oxythiamine..yeast | Campylobacter | concisus | 0,405 | 0,007 |
| PWY.7221 uanosine.ribonucleotides.de.novo.biosynthesis | Clostridiales_Family_XIII |  | 0,340 | 0,007 |
| PWY.6121..5.aminoimidazole.ribonucleotide.biosynthesis.I | Lachnospiraceae | bacterium oral taxon 96 | 0,272 | 0,007 |
| PWY.2942..L.lysine.biosynthesis.III | Prevotella | sp oral taxon 306 | 0,541 | 0,007 |
| PWY.6700..queuosine.biosynthesis.I..de.novo | Prevotella | melaninogenica | 0,378 | 0,007 |
| HSERMETANA.PWY..L.methionine.biosynthesis.III | Tannerella | sp oral taxon HOT 286 | 0,219 | 0,007 |
| GLUTORN.PWY..L.ornithine.biosynthesis.I | Lachnospiraceae | bacterium oral taxon 96 | 0,359 | 0,007 |
| ARO.PWY..chorismate.biosynthesis.I | Prevotella | histicola | 0,613 | 0,008 |
| PWY.6969..TCA.cycle.V..2.oxoglutarate.synthase. |  |  | 0,179 | 0,008 |
| PWY.6124..inosine.5..phosphate.biosynthesis.II | Oribacterium | parvum | 0,284 | 0,008 |
| PWY.5667..CDP.diacylglycerol.biosynthesis.I | Lachnospiraceae | bacterium oral taxon 96 | 0,285 | 0,008 |
| PWY0.1319..CDP.diacylglycerol.biosynthesis.II | Lachnospiraceae | bacterium oral taxon 96 | 0,285 | 0,008 |
| COA.PWY..coenzyme.A.biosynthesis.I..prokaryotic | Prevotella | jejuni | 0,575 | 0,008 |
| PWY.5837..2.carboxy.1.4.naphthoquinol.biosynthesis | Veillonella | atypica | 0,521 | 0,008 |
| PWY.6122..5.aminoimidazole.ribonucleotide.biosynthesis.II | Selenomonas | sputigena | 0,254 | 0,008 |
| PWY.6277..superpathway.of.5.aminoimidazole  .ribonucleotide.biosynthesis | Selenomonas | sputigena | 0,254 | 0,008 |
| PEPTIDOGLYCANSYN.PWY..peptidoglycan  .biosynthesis.I..meso.diaminopimelate.containing | Prevotella | jejuni | 0,684 | 0,008 |
| PYRIDNUCSYN.PWY..NAD.de.novo.biosynthesis.I..from.aspartate | Prevotella | jejuni | 0,534 | 0,008 |
| PWY.7198..pyrimidine.deoxyribonucleotides.de.novo.biosynthesis.IV | Actinomyces | sp S6 Spd3 | 0,335 | 0,008 |
| PWY.7851..coenzyme.A.biosynthesis.II..eukaryotic | Tannerella | sp oral taxon HOT 286 | 0,216 | 0,008 |
| PWY.5103..L.isoleucine.biosynthesis.III.unclassified |  |  | 0,293 | 0,008 |
| PWY.6122..5.aminoimidazole.ribonucleotide.  biosynthesis.II.unclassified |  |  | 0,149 | 0,008 |
| PWY.6277..superpathway.of.5.aminoimidazole.  ribonucleotide.biosynthesis.unclassified |  |  | 0,149 | 0,008 |
| PWY.5695..inosine.5..phosphate.degradation |  |  | 0,039 | 0,009 |
| PWY.7221 uanosine.ribonucleotides.de.novo.biosynthesis | Lachnospiraceae | bacterium oral taxon 96 | 0,305 | 0,009 |
| PWY.6823..molybdopterin.biosynthesis | Lachnospiraceae | bacterium oral taxon 96 | 0,364 | 0,009 |
| COA.PWY..coenzyme.A.biosynthesis.I..prokaryotic | Actinomyces | sp S6 Spd3 | 0,219 | 0,009 |
| PWY.2942..L.lysine.biosynthesis.III | Prevotella | jejuni | 0,558 | 0,009 |
| PWY.7228..superpathway.of uanosine.  nucleotides.de.novo.biosynthesis.I | Campylobacter | concisus | 0,384 | 0,009 |
| DTDPRHAMSYN.PWY..dTDP..beta..L.rhamnose.biosynthesis | Prevotella | sp oral taxon 306 | 0,617 | 0,009 |
| PWY.5676..acetyl.CoA.fermentation.to.butanoate.II.unclassified |  |  | 0,352 | 0,009 |
| PWY.6163..chorismate.biosynthesis.from.3.dehydroquinate | Veillonella | atypica | 0,404 | 0,009 |
| ASPASN.PWY..superpathway.of.L.aspartate.and.L  .asparagine.biosynthesis | Prevotella | jejuni | 0,656 | 0,009 |
| PWY.5103..L.isoleucine.biosynthesis.III | Veillonella | atypica | 0,467 | 0,009 |
| PWY.6700..queuosine.biosynthesis.I..de.novo | Alloprevotella | rava | 0,236 | 0,009 |
| PWY.7851..coenzyme.A.biosynthesis.II..eukaryotic | Atopobium | parvulum | 0,300 | 0,009 |
| PWY.841..superpathway.of.purine.nucleotides.de.novo.biosynthesis.I | Veillonella | atypica | 0,521 | 0,009 |
| PWY.7221 uanosine.ribonucleotides.de.novo.biosynthesis | Tannerella | sp oral taxon HOT 286 | 0,249 | 0,009 |
| PWY.6385..peptidoglycan.biosynthesis.III..mycobacteria | Tannerella | sp oral taxon HOT 286 | 0,201 | 0,009 |
| ILEUSYN.PWY..L.isoleucine.biosynthesis.I..from.threonine | Veillonella | atypica | 0,442 | 0,010 |
| TRNA.CHARGING.PWY..tRNA.charging | Prevotella | jejuni | 0,545 | 0,010 |
| P185.PWY..formaldehyde.assimilation.III  ..dihydroxyacetone.cycle..unclassified |  |  | 0,344 | 0,010 |
| PWY.3841..folate.transformations.II..plants | Actinomyces | sp oral taxon 181 | 0,310 | 0,010 |
| PWY.5188..tetrapyrrole.biosynthesis.I..from lutamate | Veillonella | atypica | 0,529 | 0,010 |
| NONMEVIPP.PWY..methylerythritol.phosphate.pathway.I | Lachnospiraceae | bacterium oral taxon 96 | 0,262 | 0,010 |
| PWY.7198..pyrimidine.deoxyribonucleotides.de.novo.biosynthesis.IV | Actinomyces | sp oral taxon 181 | 0,399 | 0,010 |
| PWY.3841..folate.transformations.II..plants | Prevotella | jejuni | 0,640 | 0,010 |
| PWY.6823..molybdopterin.biosynthesis.unclassified |  |  | 0,283 | 0,010 |
| PWY0.1319..CDP.diacylglycerol.biosynthesis.II.unclassified |  |  | 0,256 | 0,010 |
| PWY.6163..chorismate.biosynthesis.from.3.dehydroquinate | Campylobacter | concisus | 0,336 | 0,011 |
| RIBOSYN2.PWY..flavin.biosynthesis.I..bacteria.and.plants | Veillonella | atypica | 0,543 | 0,011 |
| THRESYN.PWY..superpathway.of.L.threonine.biosynthesis | Veillonella | atypica | 0,591 | 0,011 |
| PWY.6122..5.aminoimidazole.ribonucleotide.biosynthesis.II | Veillonella | atypica | 0,585 | 0,011 |
| PWY.6277..superpathway.of.5.aminoimidazole  .ribonucleotide.biosynthesis | Veillonella | atypica | 0,585 | 0,011 |
| PWY.7238..sucrose.biosynthesis.II | Actinomyces | graevenitzii | 0,390 | 0,011 |
| PWY.6121..5.aminoimidazole.ribonucleotide.biosynthesis.I | Veillonella | atypica | 0,583 | 0,011 |
| PWY.6386..UDP.N.acetylmuramoyl.pentapeptide.  biosynthesis.II..lysine.containing | Atopobium | parvulum | 0,366 | 0,011 |
| COA.PWY.1..superpathway.of.coenzyme.A.biosynthesis.III..mammals | Tannerella | sp oral taxon HOT 286 | 0,185 | 0,011 |
| BRANCHED.CHAIN.AA.SYN.PWY..superpathway  .of.branched.chain.amino.acid.biosynthesis | Veillonella | atypica | 0,417 | 0,011 |
| G.glycolysis.E.D..superpathway.of .glycolysis.and.  the.Entner.Doudoroff.pathway.unclassified |  |  | 0,227 | 0,011 |
| SER LYSYN.PWY..superpathway.of.L.serine.and lycine.biosynthesis.I | Prevotella | jejuni | 0,669 | 0,011 |
| PWY.6609..adenine.and.adenosine.salvage.III.unclassified |  |  | 0,183 | 0,011 |
| PWY.5686..UMP.biosynthesis.I | Campylobacter | concisus | 0,282 | 0,011 |
| PWY.6317..D alactose.degradation.I..Leloir.pathway | Oribacterium | asaccharolyticum | 0,185 | 0,011 |
| ARO.PWY..chorismate.biosynthesis.I | Corynebacterium | matruchotii | 0,343 | 0,011 |
| PWY.7221 uanosine.ribonucleotides.de.novo.biosynthesis | Veillonella | atypica | 0,471 | 0,011 |
| PWY.6305..superpathway.of.putrescine.biosynthesis |  |  | 0,200 | 0,012 |
| PWY.6527..stachyose.degradation.unclassified |  |  | 0,261 | 0,012 |
| PWY.702..L.methionine.biosynthesis.II | Veillonella | atypica | 0,513 | 0,012 |
| THISYNARA.PWY..superpathway.of.thiamine.  diphosphate.biosynthesis.III..eukaryotes | Clostridiales_Family_XIII |  | 0,254 | 0,012 |
| PWY.5188..tetrapyrrole.biosynthesis.I..from lutamate | Selenomonas | sputigena | 0,204 | 0,012 |
| PWY.5103..L.isoleucine.biosynthesis.III | Tannerella | sp oral taxon HOT 286 | 0,348 | 0,012 |
| P105.PWY..TCA.cycle.IV..2.oxoglutarate.decarboxylase. |  |  | 0,487 | 0,012 |
| PWY.5659 DP.mannose.biosynthesis | Prevotella | pallens | 0,537 | 0,012 |
| COLANSYN.PWY..colanic.acid.building.blocks.  biosynthesis.unclassified |  |  | 0,328 | 0,012 |
| PWY.6386..UDP.N.acetylmuramoyl.pentapeptide.  biosynthesis.II..lysine.containing..unclassified |  |  | 0,147 | 0,012 |
| ARGSYNBSUB.PWY..L.arginine.biosynthesis.II..acetyl.cycle | Lachnospiraceae | bacterium oral taxon 96 | 0,307 | 0,012 |
| UNINTEGRATED | Veillonella | atypica | 0,436 | 0,013 |
| PWY.5189..tetrapyrrole.biosynthesis.II..from lycine | Selenomonas | sputigena | 0,183 | 0,013 |
| UDPNAGSYN.PWY..UDP.N.acetyl.D lucosamine.biosynthesis.I | Selenomonas | sputigena | 0,216 | 0,013 |
| PWY0.1241..ADP.L lycero..beta..D.manno.heptose.  biosynthesis.unclassified |  |  | 0,287 | 0,013 |
| PWY.7992..superpathway.of.menaquinol.8.biosynthesis.III | Campylobacter | concisus | 0,376 | 0,013 |
| COMPLETE.ARO.PWY..superpathway.of.aromatic.  amino.acid.biosynthesis | Lachnospiraceae | bacterium oral taxon 96 | 0,258 | 0,013 |
| PWY.7221 uanosine.ribonucleotides.de.novo.biosynthesis | Oribacterium | parvum | 0,232 | 0,013 |
| PWY.6703..preQ0.biosynthesis | Veillonella | atypica | 0,399 | 0,013 |
| PWY.5667..CDP.diacylglycerol.biosynthesis.I | Clostridiales_Family_XIII |  | 0,295 | 0,013 |
| PWY0.1319..CDP.diacylglycerol.biosynthesis.II | Clostridiales_Family_XIII |  | 0,295 | 0,013 |
| PWY.6163..chorismate.biosynthesis.from.3.dehydroquinate | Corynebacterium | matruchotii | 0,330 | 0,013 |
| PWY.7199..pyrimidine.deoxyribonucleosides.salvage | Prevotella | jejuni | 0,555 | 0,013 |
| PWY.6385..peptidoglycan.biosynthesis.III..mycobacteria | Prevotella | jejuni | 0,646 | 0,014 |
| GOLPDLCAT.PWY..superpathway.of lycerol.  degradation.to.1.3.propanediol.unclassified |  |  | 0,204 | 0,014 |
| COA.PWY..coenzyme.A.biosynthesis.I..prokaryotic | Tannerella | sp oral taxon HOT 286 | 0,241 | 0,014 |
| PWY.6700..queuosine.biosynthesis.I..de.novo. |  |  | 0,053 | 0,014 |
| UNINTEGRATED | Selenomonas | sputigena | 0,405 | 0,014 |
| DTDPRHAMSYN.PWY..dTDP..beta..L.rhamnose.biosynthesis | Tannerella | sp oral taxon HOT 286 | 0,248 | 0,014 |
| UNINTEGRATED | Bifidobacterium | longum | 0,267 | 0,014 |
| HEME.BIOSYNTHESIS.II..heme.b.biosynthesis.I..aerobic | Veillonella | infantium | 0,466 | 0,014 |
| PWY.6123..inosine.5..phosphate.biosynthesis.I | Leptotrichia | sp oral taxon 498 | 0,216 | 0,014 |
| PHOSLIPSYN.PWY..superpathway.of.phospholipid.  biosynthesis.I..bacteria. |  |  | 0,068 | 0,014 |
| PWY.6387..UDP.N.acetylmuramoyl.pentapeptide.  biosynthesis.I..meso.diaminopimelate.containing | Atopobium | parvulum | 0,343 | 0,014 |
| PWY.6151..S.adenosyl.L.methionine.salvage.I | Prevotella | histicola | 0,555 | 0,014 |
| PWY.7197..pyrimidine.deoxyribonucleotide.phosphorylation | Campylobacter | concisus | 0,399 | 0,014 |
| PEPTIDOGLYCANSYN.PWY..peptidoglycan.  biosynthesis.I..meso.diaminopimelate.containing | Tannerella | sp oral taxon HOT 286 | 0,195 | 0,014 |
| PWY.5695..inosine.5..phosphate.degradation | Campylobacter | concisus | 0,441 | 0,014 |
| TRNA.CHARGING.PWY..tRNA.charging | Prevotella | sp oral taxon 306 | 0,487 | 0,015 |
| PWY.5861..superpathway.of.demethylmenaquinol  .8.biosynthesis.I.unclassified |  |  | 0,274 | 0,015 |
| SER LYSYN.PWY..superpathway.of.L.serine.and  lycine.biosynthesis.I.unclassified |  |  | 0,209 | 0,015 |
| PWY.5973..cis.vaccenate.biosynthesis | Campylobacter | showae | 0,203 | 0,015 |
| PWY.6151..S.adenosyl.L.methionine.salvage.I | Lachnospiraceae | bacterium oral taxon 96 | 0,366 | 0,015 |
| GLYCOGENSYNTH.PWY lycogen.biosynthesis.I..  from.ADP.D lucose | Actinomyces | sp S6 Spd3 | 0,324 | 0,016 |
| DTDPRHAMSYN.PWY..dTDP..beta..L.rhamnose.biosynthesis | Prevotella | histicola | 0,624 | 0,016 |
| POLYAMINSYN3.PWY..superpathway.of.polyamine.biosynthesis.II |  |  | 0,267 | 0,016 |
| RIBOSYN2.PWY..flavin.biosynthesis.I..bacteria.and.plants | Prevotella | melaninogenica | 0,315 | 0,016 |
| PWY.5100..pyruvate.fermentation.to.acetate.and.lactate.II | Veillonella | atypica | 0,503 | 0,016 |
| PWY.7851..coenzyme.A.biosynthesis.II..eukaryotic | Prevotella | sp oral taxon 306 | 0,603 | 0,016 |
| PWY.7977..L.methionine.biosynthesis.IV | Prevotella | jejuni | 0,582 | 0,017 |
| PWY.6163..chorismate.biosynthesis.from.3.dehydroquinate | Actinomyces | sp HPA0247 | 0,469 | 0,017 |
| PWY.5695..inosine.5..phosphate.degradation | Alloprevotella | rava | 0,240 | 0,017 |
| PWY0.162..superpathway.of.pyrimidine.  ribonucleotides.de.novo.biosynthesis.unclassified |  |  | 0,257 | 0,017 |
| ARO.PWY..chorismate.biosynthesis.I | Actinomyces | sp HPA0247 | 0,427 | 0,017 |
| PWY.7031..protein.N lycosylation..bacterial | Campylobacter | concisus | 0,344 | 0,017 |
| ILEUSYN.PWY..L.isoleucine.biosynthesis.I..from.threonine..unclassified |  |  | 0,272 | 0,017 |
| P42.PWY..incomplete.reductive.TCA.cycle.unclassified |  |  | 0,325 | 0,017 |
| PWY.6748..nitrate.reduction.VII..denitrification. |  |  | 0,321 | 0,018 |
| NAD.BIOSYNTHESIS.II..NAD.salvage.pathway.III  ..to.nicotinamide.riboside..unclassified |  |  | 0,293 | 0,018 |
| HEME.BIOSYNTHESIS.II..heme.b.biosynthesis.I..aerobic | Veillonella | atypica | 0,441 | 0,018 |
| VALSYN.PWY..L.valine.biosynthesis | Campylobacter | concisus | 0,404 | 0,018 |
| PWY.5686..UMP.biosynthesis.I | Prevotella | histicola | 0,499 | 0,018 |
| PWY.7790..UMP.biosynthesis.II | Prevotella | histicola | 0,499 | 0,018 |
| PWY.7791..UMP.biosynthesis.III | Prevotella | histicola | 0,499 | 0,018 |
| VALSYN.PWY..L.valine.biosynthesis | Veillonella | atypica | 0,443 | 0,019 |
| UNINTEGRATED | Alloprevotella | rava | 0,285 | 0,019 |
| PWY.7761..NAD.salvage.pathway.II..PNC.IV.cycle..unclassified |  |  | 0,356 | 0,019 |
| P164.PWY..purine.nucleobases.degradation.I..anaerobic. |  |  | 0,157 | 0,019 |
| RIBOSYN2.PWY..flavin.biosynthesis.I..bacteria.and.plants | Clostridiales_Family_XIII |  | 0,186 | 0,020 |
| PWY.6387..UDP.N.acetylmuramoyl.pentapeptide.  biosynthesis.I..meso.diaminopimelate.containing | Tannerella | sp oral taxon HOT 286 | 0,194 | 0,020 |
| PWY.6527..stachyose.degradation |  |  | 0,100 | 0,020 |
| PWY.2942..L.lysine.biosynthesis.III |  |  | 0,033 | 0,020 |
| PWY.5855..ubiquinol.7.biosynthesis..early.decarboxylation | Neisseria | macacae | 0,309 | 0,021 |
| PWY.6147..6.hydroxymethyl.dihydropterin.diphosphate.  biosynthesis.I.unclassified |  |  | 0,240 | 0,021 |
| PANTOSYN.PWY..superpathway.of.coenzyme.A  .biosynthesis.I..bacteria..unclassified |  |  | 0,234 | 0,021 |
| PWY.7977..L.methionine.biosynthesis.IV |  |  | 0,092 | 0,021 |
| OANTIGEN.PWY..O.antigen.building.blocks.biosynthesis..  E..coli..unclassified |  |  | 0,180 | 0,021 |
| PWY.8178..pentose.phosphate.pathway..non.oxidative.  branch..II.unclassified |  |  | 0,176 | 0,021 |
| PWY.6121..5.aminoimidazole.ribonucleotide.biosynthesis.I | Selenomonas | sputigena | 0,211 | 0,022 |
| PWY.7238..sucrose.biosynthesis.II | Oribacterium | asaccharolyticum | 0,340 | 0,022 |
| VALSYN.PWY..L.valine.biosynthesis | Tannerella | sp oral taxon HOT 286 | 0,326 | 0,022 |
| PWY.7208..superpathway.of.pyrimidine.nucleobases.salvage | Actinomyces | sp HPA0247 | 0,505 | 0,023 |
| UNINTEGRATED | Actinomyces | graevenitzii | 0,405 | 0,023 |
| DTDPRHAMSYN.PWY..dTDP..beta..L.rhamnose.biosynthesis | Megasphaera | micronuciformis | 0,344 | 0,023 |
| PWY.6122..5.aminoimidazole.ribonucleotide.biosynthesis.II | Oribacterium | asaccharolyticum | 0,332 | 0,023 |
| PWY.6277..superpathway.of.5.aminoimidazole  .ribonucleotide.biosynthesis | Oribacterium | asaccharolyticum | 0,332 | 0,023 |
| HISDEG.PWY..L.histidine.degradation.I | Tannerella | sp oral taxon HOT 286 | 0,265 | 0,023 |
| HISDEG.PWY..L.histidine.degradation.I | Alloprevotella | rava | 0,291 | 0,023 |
| GLUTORN.PWY..L.ornithine.biosynthesis.I | Leptotrichia | sp oral taxon 498 | 0,233 | 0,023 |
| PWY.6700..queuosine.biosynthesis.I..de.novo | Oribacterium | parvum | 0,188 | 0,023 |
| X1CMET2.PWY..folate.transformations.III..E..coli | Prevotella | histicola | 0,562 | 0,023 |
| PWY.7199..pyrimidine.deoxyribonucleosides.salvage | Prevotella | histicola | 0,533 | 0,023 |
| ASPASN.PWY..superpathway.of.L.aspartate.and.L.asparagine.biosynthesis | Prevotella | sp oral taxon 306 | 0,510 | 0,023 |
| PWY.6284..superpathway.of.unsaturated.fatty.acids  .biosynthesis..E..coli..unclassified |  |  | 0,367 | 0,023 |
| PWY.6163..chorismate.biosynthesis.from.3.dehydroquinate | Selenomonas | sputigena | 0,215 | 0,024 |
| SER LYSYN.PWY..superpathway.of.L.serine.and  lycine.biosynthesis.I | Prevotella | sp oral taxon 306 | 0,582 | 0,024 |
| THISYNARA.PWY..superpathway.of.thiamine.  diphosphate.biosynthesis.III..eukaryotes | Prevotella | melaninogenica | 0,327 | 0,024 |
| ILEUSYN.PWY..L.isoleucine.biosynthesis.I..from.threonine | Actinomyces | sp S6 Spd3 | 0,340 | 0,024 |
| PWY.6151..S.adenosyl.L.methionine.salvage.I | Atopobium | parvulum | 0,369 | 0,025 |
| PWY.841..superpathway.of.purine.nucleotides.  de.novo.biosynthesis.I.unclassified |  |  | 0,190 | 0,025 |
| PWY.7851..coenzyme.A.biosynthesis.II..eukaryotic | Prevotella | histicola | 0,528 | 0,025 |
| PWY.7234..inosine.5..phosphate.biosynthesis.III.unclassified |  |  | 0,237 | 0,025 |
| HEMESYN2.PWY..heme.b.biosynthesis.II..oxygen.independent | Veillonella | atypica | 0,472 | 0,026 |
| PWY.5005..biotin.biosynthesis.II | Veillonella | dispar | 0,439 | 0,026 |
| PWY.6823..molybdopterin.biosynthesis | Selenomonas | sputigena | 0,199 | 0,026 |
| PWY.6609..adenine.and.adenosine.salvage.III | Selenomonas | sputigena | 0,182 | 0,026 |
| UNINTEGRATED | Tannerella | sp oral taxon HOT 286 | 0,357 | 0,026 |
| PWY.7663 ondoate.biosynthesis..anaerobic | Prevotella | sp oral taxon 306 | 0,577 | 0,026 |
| NONMEVIPP.PWY..methylerythritol.phosphate.pathway.I | Clostridiales_Family_XIII |  | 0,194 | 0,026 |
| PWY66.429..fatty.acid.biosynthesis.initiation..mitochondria | Lachnospiraceae | bacterium oral taxon 96 | 0,320 | 0,026 |
| PWY.6606 uanosine.nucleotides.degradation.II | Oribacterium | sinus | 0,305 | 0,026 |
| PWY.6823..molybdopterin.biosynthesis | Oribacterium | parvum | 0,211 | 0,027 |
| PWY.6147..6.hydroxymethyl.dihydropterin.diphosphate.biosynthesis.I | Prevotella | melaninogenica | 0,344 | 0,027 |
| PWY.5103..L.isoleucine.biosynthesis.III | Actinomyces | sp S6 Spd3 | 0,350 | 0,027 |
| PWY.6151..S.adenosyl.L.methionine.salvage.I | Actinomyces | sp S6 Spd3 | 0,317 | 0,027 |
| DTDPRHAMSYN.PWY..dTDP..beta..L.rhamnose.biosynthesis | Veillonella | atypica | 0,611 | 0,027 |
| PWY.6803..phosphatidylcholine.acyl.editing | Campylobacter | showae | 0,189 | 0,027 |
| PWY.6151..S.adenosyl.L.methionine.salvage.I | Oribacterium | parvum | 0,289 | 0,027 |
| PWY.3001..superpathway.of.L.isoleucine.biosynthesis.I.unclassified |  |  | 0,258 | 0,027 |
| VALSYN.PWY..L.valine.biosynthesis | Campylobacter | showae | 0,220 | 0,027 |
| DAPLYSINESYN.PWY..L.lysine.biosynthesis.I | Actinomyces | sp HPA0247 | 0,431 | 0,027 |
| PWY.7977..L.methionine.biosynthesis.IV | Prevotella | melaninogenica | 0,323 | 0,027 |
| PWY.6317..D alactose.degradation.I..Leloir.pathway..unclassified |  |  | 0,180 | 0,027 |
| PWY.6125..superpathway.of uanosine.nucleotides.de.novo.biosynthesis.II | Actinomyces | sp HPA0247 | 0,465 | 0,028 |
| PWY0.1586..peptidoglycan.maturation..meso.diaminopimelate.containing | Veillonella | atypica | 0,524 | 0,028 |
| PWY.7400..L.arginine.biosynthesis.IV..archaebacteria..unclassified |  |  | 0,197 | 0,028 |
| UNINTEGRATED | Campylobacter | concisus | 0,403 | 0,028 |
| PWY.7228..superpathway.of uanosine.nucleotides.de.novo.biosynthesis.I | Actinomyces | sp S6 Spd3 | 0,337 | 0,028 |
| PWY.5667..CDP.diacylglycerol.biosynthesis.I | Oribacterium | parvum | 0,208 | 0,028 |
| PWY0.1319..CDP.diacylglycerol.biosynthesis.II | Oribacterium | parvum | 0,208 | 0,028 |
| X1CMET2.PWY..folate.transformations.III..E..coli | Prevotella | melaninogenica | 0,343 | 0,028 |
| GLYCOGENSYNTH.PWY lycogen.biosynthesis.I..from.ADP.D lucose | Selenomonas | sputigena | 0,169 | 0,028 |
| POLYAMSYN.PWY..superpathway.of.polyamine.biosynthesis.I |  |  | 0,328 | 0,028 |
| PWY4LZ.257..superpathway.of.fermentation..Chlamydomonas  .reinhardtii..unclassified |  |  | 0,174 | 0,028 |
| THRESYN.PWY..superpathway.of.L.threonine.biosynthesis.  unclassified |  |  | 0,177 | 0,029 |
| LACTOSECAT.PWY..lactose.and alactose.degradation.I | Leptotrichia | wadei | 0,386 | 0,029 |
| PWY.5973..cis.vaccenate.biosynthesis | Prevotella | sp oral taxon 306 | 0,554 | 0,029 |
| PWY.6282..palmitoleate.biosynthesis.I..from..5Z..dodec.5.enoate | Campylobacter | showae | 0,168 | 0,029 |
| ANAG.glycolysis.PWY.glycolysis.III..from lucose | Prevotella | histicola | 0,548 | 0,029 |
| PWY0.862...5Z..dodecenoate.biosynthesis.I | Campylobacter | showae | 0,172 | 0,030 |
| PWY.7238..sucrose.biosynthesis.II.unclassified |  |  | 0,163 | 0,030 |
| TRNA.CHARGING.PWY..tRNA.charging | Prevotella | melaninogenica | 0,337 | 0,030 |
| PYRIDNUCSYN.PWY..NAD.de.novo.biosynthesis.I..from.aspartate | Prevotella | histicola | 0,486 | 0,030 |
| PWY.7664..oleate.biosynthesis.IV..anaerobic | Campylobacter | showae | 0,168 | 0,030 |
| PWY.3841..folate.transformations.II..plants. |  |  | 0,029 | 0,030 |
| PWY.6527..stachyose.degradation | Oribacterium | parvum | 0,172 | 0,030 |
| RIBOSYN2.PWY..flavin.biosynthesis.I..bacteria.and.plants | Prevotella | jejuni | 0,401 | 0,030 |
| UNINTEGRATED | Prevotella | histicola | 0,470 | 0,030 |
| UNINTEGRATED | Rothia | dentocariosa | -0,347 | 0,030 |
| LACTOSECAT.PWY..lactose.and alactose.degradation.I |  |  | -0,117 | 0,030 |
| PWY.6608 uanosine.nucleotides.degradation.III |  |  | 0,122 | 0,030 |
| FASYN.ELONG.PWY..fatty.acid.elongation....saturated | Campylobacter | showae | 0,164 | 0,030 |
| ARG.POLYAMINE.SYN..superpathway.of.arginine  .and.polyamine.biosynthesis |  |  | 0,312 | 0,030 |
| PWY.7282..4.amino.2.methyl.5.diphosphomethylpyrimidine  .biosynthesis.II.unclassified |  |  | 0,254 | 0,030 |
| SER LYSYN.PWY..superpathway.of.L.serine.and lycine.biosynthesis.I | Campylobacter | showae | 0,269 | 0,030 |
| DTDPRHAMSYN.PWY..dTDP..beta..L.rhamnose.biosynthesis | Selenomonas | sputigena | 0,190 | 0,031 |
| PWY.6124..inosine.5..phosphate.biosynthesis.II | Oribacterium | asaccharolyticum | 0,310 | 0,031 |
| PWY.5941 lycogen.degradation.II | Actinomyces | sp S6 Spd3 | 0,236 | 0,031 |
| PWY.6936..seleno.amino.acid.biosynthesis..plants | Campylobacter | concisus | 0,286 | 0,031 |
| PWY.3841..folate.transformations.II..plants | Actinomyces | sp S6 Spd3 | 0,285 | 0,031 |
| PANTOSYN.PWY..superpathway.of.coenzyme.A.biosynthesis.I..bacteria | Tannerella | sp oral taxon HOT 286 | 0,176 | 0,031 |
| COA.PWY..coenzyme.A.biosynthesis.I..prokaryotic | Prevotella | histicola | 0,542 | 0,032 |
| PWY.3841..folate.transformations.II..plants | Prevotella | histicola | 0,571 | 0,032 |
| PWY.7977..L.methionine.biosynthesis.IV | Tannerella | sp oral taxon HOT 286 | 0,169 | 0,032 |
| BRANCHED.CHAIN.AA.SYN.PWY..superpathway.of.  branched.chain.amino.acid.biosynthesis | Actinomyces | sp S6 Spd3 | 0,297 | 0,032 |
| PWY.6703..preQ0.biosynthesis |  |  | 0,051 | 0,032 |
| PWY.7221 uanosine.ribonucleotides.de.novo.biosynthesis | Oribacterium | sinus | 0,364 | 0,032 |
| COA.PWY.1..superpathway.of.coenzyme.A.biosynthesis.III..mammals | Clostridiales_Family_XIII |  | 0,181 | 0,033 |
| PWY.5097..L.lysine.biosynthesis.VI | Prevotella | histicola | 0,490 | 0,033 |
| PWY.6895..superpathway.of.thiamine.diphosphate.biosynthesis.II |  |  | 0,317 | 0,033 |
| VALSYN.PWY..L.valine.biosynthesis | Oribacterium | parvum | 0,316 | 0,033 |
| PWY.5989..stearate.biosynthesis.II..bacteria.and.plants | Campylobacter | showae | 0,162 | 0,033 |
| PWY.7197..pyrimidine.deoxyribonucleotide.phosphorylation | Actinomyces | sp HPA0247 | 0,436 | 0,033 |
| NONMEVIPP.PWY..methylerythritol.phosphate.pathway.I | Prevotella | melaninogenica | 0,333 | 0,033 |
| PWY.7111..pyruvate.fermentation.to.isobutanol..engineered | Oribacterium | parvum | 0,303 | 0,034 |
| PWY.6285..superpathway.of.fatty.acids.biosynthesis..E..coli..unclassified |  |  | 0,195 | 0,034 |
| PWY.6936..seleno.amino.acid.biosynthesis..plants | Actinomyces | sp S6 Spd3 | 0,327 | 0,035 |
| PWY.7953..UDP.N.acetylmuramoyl.pentapeptide.  biosynthesis.III..meso.diaminopimelate.containing | Tannerella | sp oral taxon HOT 286 | 0,164 | 0,035 |
| PWY.7858...5Z..dodecenoate.biosynthesis.II |  |  | -0,201 | 0,035 |
| VALSYN.PWY..L.valine.biosynthesis | Prevotella | histicola | 0,422 | 0,035 |
| PWY0.1296..purine.ribonucleosides.degradation | Selenomonas | sputigena | 0,176 | 0,036 |
| PWY.6897..thiamine.diphosphate.salvage.II.unclassified |  |  | 0,218 | 0,036 |
| PWY.8187..L.arginine.degradation.XIII..reductive.Stickland.reaction | Actinomyces | sp S6 Spd3 | 0,224 | 0,036 |
| PWY.6700..queuosine.biosynthesis.I..de.novo | Campylobacter | concisus | 0,316 | 0,036 |
| PWY.702..L.methionine.biosynthesis.II |  |  | -0,066 | 0,036 |
| PWY.5837..2.carboxy.1.4.naphthoquinol.biosynthesis.unclassified |  |  | 0,299 | 0,037 |
| PWY.5838..superpathway.of.menaquinol.8.biosynthesis.I |  |  | 0,195 | 0,037 |
| UDPNAGSYN.PWY..UDP.N.acetyl.D lucosamine.biosynthesis.I | Corynebacterium | matruchotii | 0,333 | 0,037 |
| PWY.5030..L.histidine.degradation.III | Tannerella | sp oral taxon HOT 286 | 0,312 | 0,038 |
| PWY.7220..adenosine.deoxyribonucleotides.de.novo.biosynthesis.II | Actinomyces | sp S6 Spd3 | 0,307 | 0,038 |
| PWY.7222 uanosine.deoxyribonucleotides.de.novo.biosynthesis.II | Actinomyces | sp S6 Spd3 | 0,307 | 0,038 |
| PWY.6122..5.aminoimidazole.ribonucleotide.biosynthesis.II | Oribacterium | parvum | 0,339 | 0,039 |
| PWY.6277..superpathway.of.5.aminoimidazole.ribonucleotide.biosynthesis | Oribacterium | parvum | 0,339 | 0,039 |
| DTDPRHAMSYN.PWY..dTDP..beta..L.rhamnose.biosynthesis.unclassified |  |  | 0,175 | 0,039 |
| PWY.5030..L.histidine.degradation.III | Prevotella | histicola | 0,516 | 0,039 |
| PWY.7663 ondoate.biosynthesis..anaerobic | Campylobacter | showae | 0,237 | 0,039 |
| PWY.5861..superpathway.of.demethylmenaquinol.8.biosynthesis.I |  |  | 0,204 | 0,039 |
| PWY.2942..L.lysine.biosynthesis.III | Prevotella | histicola | 0,583 | 0,040 |
| PWY1ZNC.1..assimilatory.sulfate.reduction.IV |  |  | 0,419 | 0,040 |
| PWY.5686..UMP.biosynthesis.I | Prevotella | melaninogenica | 0,292 | 0,040 |
| PWY.7790..UMP.biosynthesis.II | Prevotella | melaninogenica | 0,292 | 0,040 |
| PWY.7791..UMP.biosynthesis.III | Prevotella | melaninogenica | 0,292 | 0,040 |
| P161.PWY..acetylene.degradation..anaerobic | Streptococcus | oralis | 0,446 | 0,040 |
| PWY0.1477..ethanolamine.utilization | Streptococcus | oralis | 0,446 | 0,040 |
| TRNA.CHARGING.PWY..tRNA.charging | Tannerella | sp oral taxon HOT 286 | 0,155 | 0,040 |
| POLYISOPRENSYN.PWY..polyisoprenoid.biosynthesis..E..coli. |  |  | -0,072 | 0,040 |
| PWY.6163..chorismate.biosynthesis.from.3.dehydroquinate | Prevotella | histicola | 0,465 | 0,041 |
| PWY.7229..superpathway.of.adenosine.nucleotides.de.novo.biosynthesis.I | Veillonella | dispar | 0,381 | 0,041 |
| PWY.7851..coenzyme.A.biosynthesis.II..eukaryotic | Clostridiales_Family_XIII |  | 0,186 | 0,042 |
| PWY.7238..sucrose.biosynthesis.II | Actinomyces | sp S6 Spd3 | 0,233 | 0,042 |
| PWY0.862...5Z..dodecenoate.biosynthesis.I | Aggregatibacter | sp oral taxon 458 | 0,340 | 0,042 |
| PWY.7238..sucrose.biosynthesis.II | Oribacterium | parvum | 0,317 | 0,042 |
| PWY.6700..queuosine.biosynthesis.I..de.novo | Selenomonas | sputigena | 0,153 | 0,042 |
| NONMEVIPP.PWY..methylerythritol.phosphate.pathway.I | Prevotella | histicola | 0,467 | 0,042 |
| COA.PWY..coenzyme.A.biosynthesis.I..prokaryotic | Prevotella | melaninogenica | 0,241 | 0,042 |
| PWY.821..superpathway.of.sulfur.amino.acid.biosynthesis.  .Saccharomyces.cerevisiae. |  |  | 0,392 | 0,044 |
| X1CMET2.PWY..folate.transformations.III..E..coli. |  |  | 0,065 | 0,044 |
| VALSYN.PWY..L.valine.biosynthesis | Rothia | dentocariosa | -0,375 | 0,044 |
| P41.PWY..pyruvate.fermentation.to.acetate.and..S..lactate.I | Neisseria | sp HMSC064E01 | 0,285 | 0,045 |
| PWY.5100..pyruvate.fermentation.to.acetate.and.lactate.II | Neisseria | sp HMSC064E01 | 0,285 | 0,045 |
| PWY.7031..protein.N lycosylation..bacterial. |  |  | 0,297 | 0,045 |
| PWY.5659 DP.mannose.biosynthesis.unclassified |  |  | 0,264 | 0,045 |
| PWY.7221 uanosine.ribonucleotides.de.novo.biosynthesis |  |  | 0,026 | 0,045 |
| PWY.5667..CDP.diacylglycerol.biosynthesis.I | Prevotella | melaninogenica | 0,349 | 0,046 |
| PWY0.1319..CDP.diacylglycerol.biosynthesis.II | Prevotella | melaninogenica | 0,349 | 0,046 |
| PWY.7221 uanosine.ribonucleotides.de.novo.biosynthesis | Prevotella | histicola | 0,649 | 0,046 |
| ARO.PWY..chorismate.biosynthesis.I | Prevotella | melaninogenica | 0,294 | 0,046 |
| ANAG.glycolysis.PWY.glycolysis.III..from lucose. |  |  | 0,023 | 0,046 |
| BIOTIN.BIOSYNTHESIS.PWY..biotin.biosynthesis.I.unclassified |  |  | 0,319 | 0,047 |
| PWY.5130..2.oxobutanoate.degradation.I | Veillonella | infantium | 0,490 | 0,048 |
| PWY.7953..UDP.N.acetylmuramoyl.pentapeptide.biosynthesis.  III..meso.diaminopimelate.containing | Prevotella | histicola | 0,476 | 0,049 |
